# Global Identification, Classification, and Expression Analysis of MAPKKK genes: Functional Characterization of *MdRaf5* Reveals Evolution and Drought-Responsive Profile in Apple

**DOI:** 10.1038/s41598-017-13627-2

**Published:** 2017-10-18

**Authors:** Meihong Sun, Yang Xu, Jinguang Huang, Zesheng Jiang, Huairui Shu, Huasen Wang, Shizhong Zhang

**Affiliations:** 1 State Key Laboratory of Crop Biology, Shandong Agricultural University, Tai’an, Shandong 271018 P.R. China; 2 Zhejiang Agriculture & Forestry University, Linan, Zhejiang 311300 P.R. China

## Abstract

Mitogen-activated protein kinase kinase kinases (MAPKKKs) are pivotal components of Mitogen-activated protein kinase (MAPK) cascades, which play a significant role in many biological processes. Although genome-wide analysis of MAPKKKs has been conducted in many species, extant results in apple are scarce. In this study, a total of 72 putative MdMAPKKKs in Raf-like group, 11 in ZIK-like group and 37 in MEEK were identified in apple firstly. Predicted MdMAPKKKs were located in 17 chromosomes with diverse densities, and there was a high-level of conservation in and among the evolutionary groups. Encouragingly, transcripts of 12 selected MdMAPKKKs were expressed in at least one of the tested tissues, indicating that MdMAPKKKs might participate in various physiological and developmental processes in apple. Moreover, they were found to respond to drought stress in roots and leaves, which suggested a possible conserved response to drought stress in different species. Overexpression of *MdRaf5* resulted in a hyposensitivity to drought stress, which was at least partially due to the regulation of stomatal closure and transpiration rates. To the best of our knowledge, this is the first genome-wide functional analysis of the MdMAPKKK genes in apple, and it provides valuable information for understanding MdMAPKKKs signals and their putative functions.

## Introduction

Protein kinases (PK), the most prominent superfamily in the elaborate matrix of signal transduction proteins, allow cells of eukaryotic organisms to grow and development in a coordinated manner^1–3^. Mitogen-activated protein kinase (MAPK) has formed a PK member, which is evolutionarily conserved and fundamental signal transduction pathways that plays roles downstream of various receptors/sensors that transduces extracellular stimuli into intracellular responses in eukaryotes^4^. The signal transduction modules of MAPK play a significant role in the plant’s growth, development, and regulation of various abiotic and biotic stresses, such as drought, low temperature, high salt, mechanical damage, osmotic stress, oxidative stress and pathogen infection^5–8^.

To date, MAPK’s cascades have been identified in a variety of plants after the completion of the whole genome sequence. A total of 20 MAPK, 10 MAPKK, and 80 MAPKKK genes have been reported in the *Arabidopsis thaliana* genome, whereas the rice (*Oryza sativa*) genome contains 17 MAPK, 8 MAPKK, and 75 MAPKKK genes^6,9^. Recent studies demonstrate that 19 MAPK, 9 MAPKK, and 74 MAPKKK genes can be found in maize (*Zea mays L*), and the tomato (*Lycopersicon esculentum Mill*.) genome-sequencing project has discovered 16 MAPKs, 6 MAPKKs and 89 MAPKKKs^10,11^. 14 MAPKs, 6 MAPKKs and 59 MAPKKKs have recently been identified in cucumber (*Cucumis sativus L*.); and 28 MAPKs, 11 MAPKKs and 78 MAPKKKs are present in the cotton (*Gossypium spp*) genome^12,13^. MAPKKKs activated MAPKKs, themselves, by phosphorylating the conserved serine and/or threonine residues in their T-loop, MAPKKs in turn activate a specific MAPK^4,12,14^. Compared to MAPKs and MAPKKs, the MAPKKKs have more members and greater variety in primary structures and domain composition^15^. They consist of long N- or C- terminal regions that can be subdivided into MEKK, Raf-like and ZIK-like groups according to their sequence alignment^7,16^. Phylogenetic analysis of the MAPKKK genes in various species shows that the diversity exists in plants. There are 46 MAPKKKs from maize, 43 from rice, 27 from grapevines, and 48 from Arabidopsis in the Raf group; the MEKK group consists of 22 maize, 22 rice, 9 grapevine, and 21 Arabidopsis MAPKKKs; but only 6 MAPKKKs from maize, 10 from rice, 9 from grapevines, and 11 from Arabidopsis are grouped into the ZIK group^7,9,11,17^. Structural domains of MAPKKKs in Arabidopsis, rice and cucumber show that most Raf proteins have a C-terminal kinase domain and a long N-terminal regulatory domain. In contrast, members of the ZIK group have the N-terminal kinase domain, whilst members of the MEKK group have less conserved kinase domain which consist in either N- or C-terminals or lie in the central part of the protein^9,13,18^.

However, the scarcity of information regarding the MAPKKKs in apple (*Malus domestica*) presents a bottleneck to investigating MAPK cascades in this economically important crop. The apple tree is one of the fruit trees widely cultivated by farmers in China, and it is also the most important and economical woody plant throughout the temperate zones^19^. Genome-wide analysis of the RING finger gene family^20^, DREB gene family^21^, dehydrin gene family^22^, Hsf gene family^23^, MAPK and MAPKK genes^24^ have been reported in apple. However, there is little genome-wide information currently available on apple MAPKKK genes. The apple genome has been sequenced in recent years, offering an excellent opportunity for the analysis of MAPKKK genes across the genome^25^.

MAPKKKs play a significant role in distinct biological and physiological processes, and they have potential applications for the development of stress-tolerant transgenic plants. The present study marks the first systematic analysis of the apple MdMAPKKK genes. We predicted the chromosome location and gene structure of putative MAPKKKs through genome-wide surveys of apple genomic sequences. Additionally, putative MAPKKKs were subjected to phylogenetic analyses with their Arabidopsis counterparts to identify gene orthologues and clusters of orthologous groups for further functional characterization. Furthermore, qRT-PCR was performed to determine expression patterns of selected MdMAPKKK genes. Moreover, we were interested in MdMAPKKK genes related to drought stresses; thus, we identified drought-responsive genes using transgenic plants. To the best of our knowledge, the current study is the first genome-wide analysis of the MAPKKK genes in apple. Information gained through this study may contribute to our understanding of the classification and putative functions of MdMAPKKKs in apple.

## Results

### Genome-wide identification of MAPKKK genes in apple

To identify members of the MAPKKK genes in apple, we employed diverse bioinformatic methods to gather large amounts of bioinformation to analyze. A total of 123 putative MAPKKK gene members were identified and measured in the complete apple genome. All 123 MAPKKKs had conserved protein kinase domains, which was the backbone of the MAPKKKs according to domain analysis (Fig. S1). To date, 80, 75 and 74 MAPKKK members had been identified in Arabidopsis, rice and maize, respectively^7,9,10^. The total number of the MAPKKKs in apple was approximately 1.5-fold higher than that discovered in others, suggesting the possible expansion of MAPKKKs in apple.

Specific information concerning apple MAPKKK genes was identified in the present study and listed in Supplementary Table S1. The open reading frame (ORF) of MdMPKKK ranged from 1029 bp (MdRaf23) to 6114 bp (MdRaf37), with an average ORF length of 2022 bp. The length of measured MdMAPKKKs ranged from 342 (MdRaf23) to 2037 (MdRaf37) amino acids (aa), with an average of 673 aa. The molecular weight and pIs of MdMAPKKKs ranged from 38,276.04 Da (MdMAPKKK17) to 225,516.9 Da (MdRaf37) and from 4.64 (MdMPKKK20/21) to 9.46 (MdRaf64), respectively. These results indicated that MdMAPKKKs acted as a highly conserved and expanded members in the apple genome.

### Phylogenetic analysis of MAPKKKs

To evaluate evolutionary relationships between MAPKKKs in apple and Arabidopsis, full-length amino acid sequences of 123 MdMAPKKKs and 80 AtMAPKKKs were subjected to multiple sequence alignment with the MEGA5 program. The multiple sequence alignment file was subsequently used to construct an unrooted phylogenetic tree using the neighbour-joining (NJ) method. As illustrated in Fig. S1, the phylogenetic tree of Raf-like, ZIK-like and MEKK groups were constructed according to comprehensive analysis of previous study^3,26,27^. There were 72, 37 and 11 MAPKKKs in Raf-like, MEKK and ZIK-like groups, respectively, whereas the number of genes in each group is similar to Arabidopsis, rice and maize but quite different in apple (Supplementary Table S2). Then we designated 123 MdMAPKKKs firstly in apple based on their major categories and location on the genome according to the previous study (Supplementary Table S1).

In addition, to examine evolutionary relationships of MdMAPKKKs intuitively, a phylogenetic tree was constructed from alignments of full MdMAPKKKs amino acid sequences. The phylogenetic tree has forty MdMAPKKK paralogous gene pairs, which include 86 members; these gene pairs represent approximately 70% of MAPKKK genes in apple (Fig. S2). In addition, thirty MdMAPKKK gene sister pairs were discovered; these were marked in red, indicating strong bootstrap support (>90%). MEKK contained 10 pairs; Raf constituted the largest clade, containing 18 sister pairs; and there were only two pairs in the ZIK groups (Fig. S2). Earlier studies have shown that ZmMAPKKK paralogous gene pairs represent 52% of the MAPKKKs in the phylogenetic tree of maize, suggesting that the apple MAPKKKs which have forty MdMAPKKK paralogous gene pairs may have undergone many more duplications than maize during its evolutionary history^10^.

To further analyse evolutionary relationships of MAPKKKs in *Rosaceae*, MAPKKKs were identified from peach, strawberry and pear using the same methods. Results indicated high similarity amongst *Rosaceae* MAPKKKs (Fig. S3). Furthermore, MAPKKK members showed higher similarity between apple and pear than between peach and strawberry (Fig. S3). The divergence between pear and apple occurred at approximately 5.4–21.5 million years ago, whilst the divergence from strawberry and peach occurred 65–55 million years ago^28^.

### Gene structure and conserved domain analysis in MdMAPKKKs

The structural analysis yielded valuable information concerning duplication events through interpretation of phylogenetic relationships in the genes. Based on multiple alignments of MAPKKK genes, we derived the typical structural features amongst MdMAPKKKs. In the MdMAPKKKs, the number of exons changed from 1 to 24 (Fig. S2 and Supplementary Table S1). There were 8–19 exons in most apple MEKKs, whereas 10 MEKK genes had only one exon (Fig. S2c). All members of the Raf and ZIK groups possessed 1–24 exons and 1–13 exons, respectively (Fig. S2a,b and Supplementary Table S1). The data also revealed that *MdRaf57* has the largest number of exons in apple MAPKKK genes, consistent with the exon number of its orthologue in Arabidopsis. Furthermore, *MdMAPKKK19*, *MdMAPKKK25* and *MdMAPKKK26* had longer introns, similar to two ZIK genes (*MdZIK1/3*) and six members of the Raf (*MdRaf21/29/31/37/57/71*) (Fig. S2b). Therefore, most members of the same groups shared a similar exon/intron structure and length. Based on genome-wide analysis for other plants, we discovered that most maize MEKKs have 8–17 exons, and all members of the Raf and ZIK groups possess 2–17 and 7–9 exons, respectively^10^. The greater variety of exon number in each group in apple than in maize supported their possible complexity in evolution.

**Figure 1:**
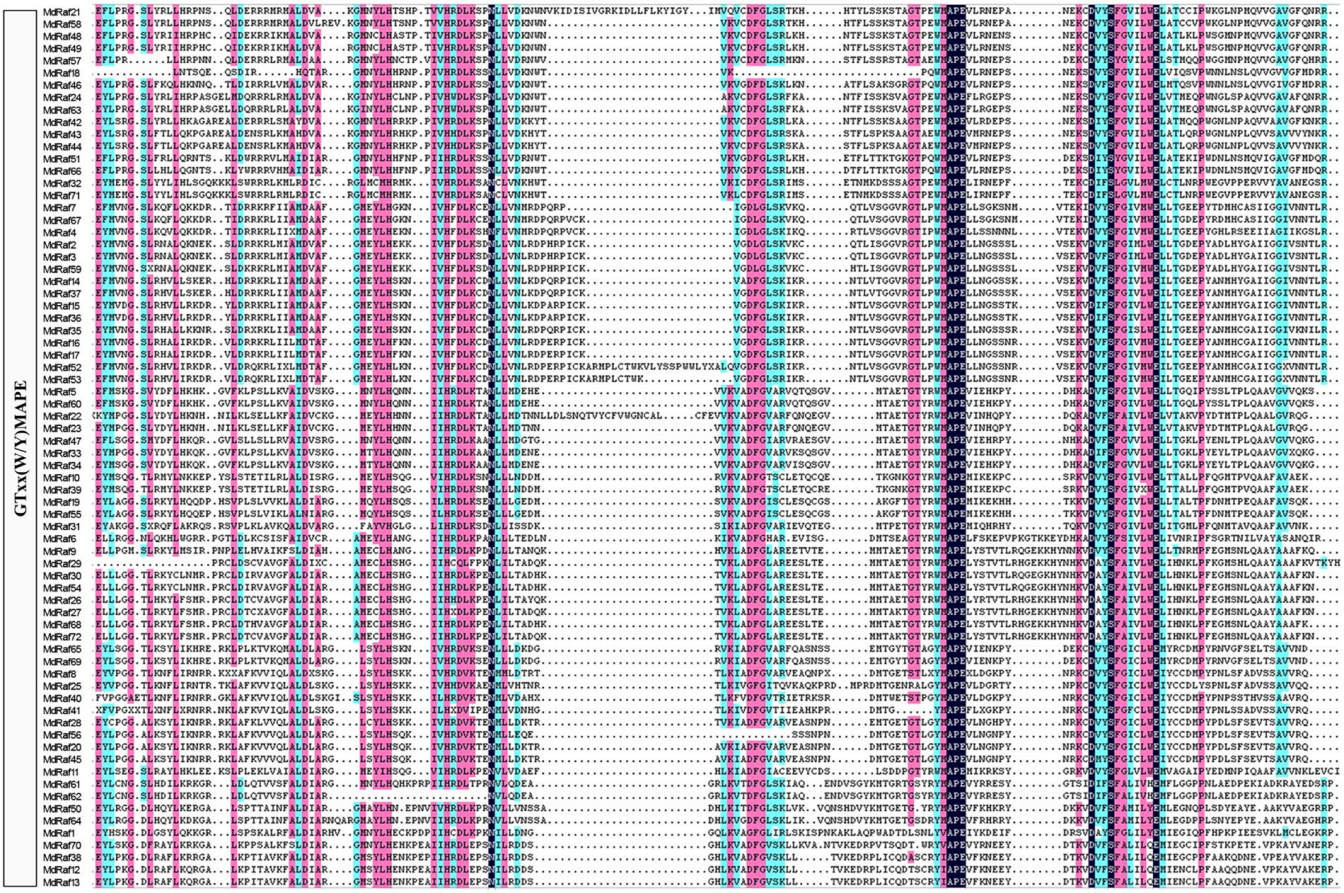
Multiple sequence alignment of Raf-like group from apple. The highlighted part shows the conservative motif.

**Figure 2:**
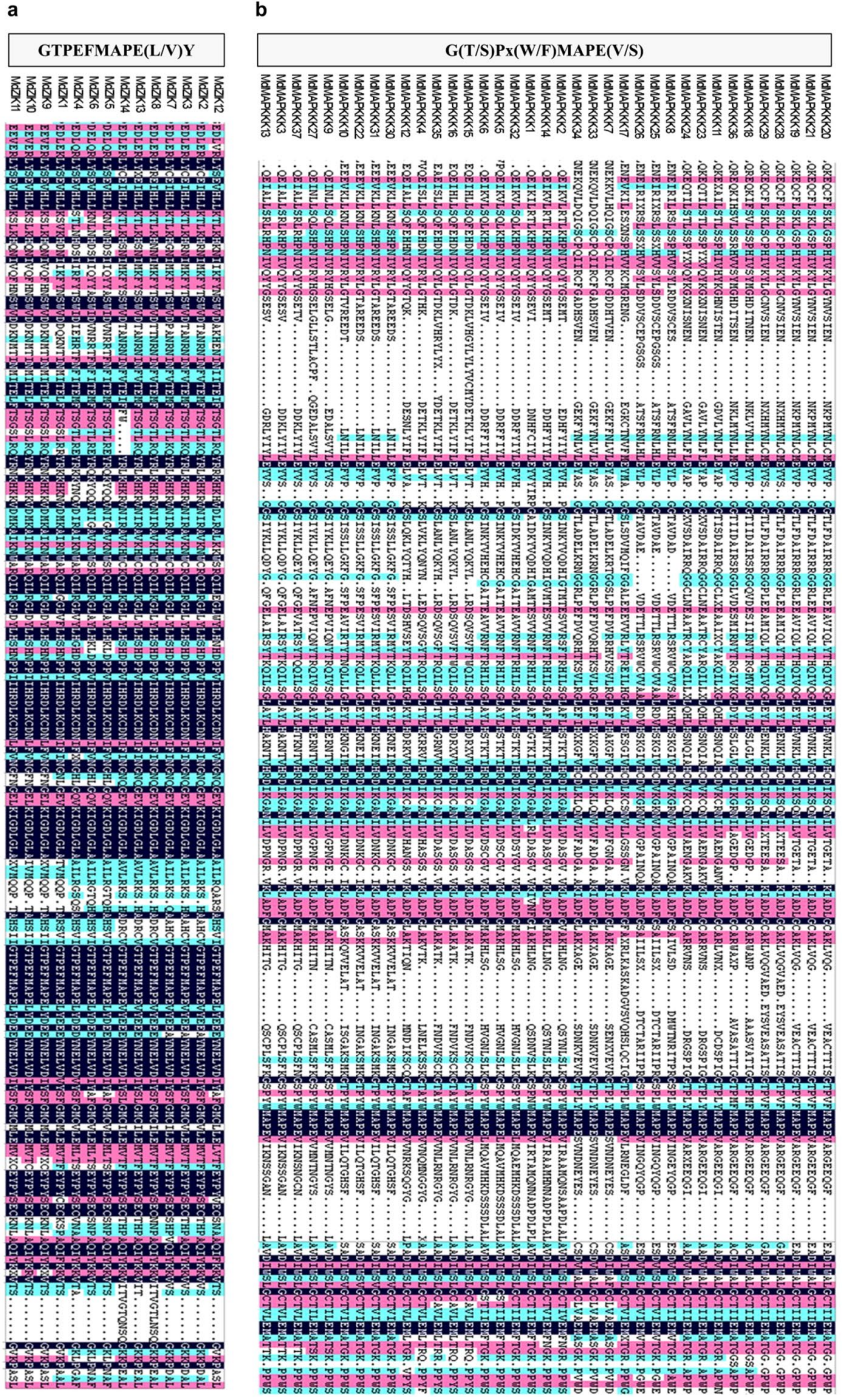
Multiple sequence alignment of (**a**) ZIK-like, (**b**) MEKK groups from apple. The highlighted part shows the conservative motif.

Protein multiple sequence alignments for Arabidopsis and apple revealed the conserved amino acid residues amongst MAPKKKs. As shown in Fig. S1, all of the MAPKKKs contained the kinase domain. Almost all Raf members had extended N-terminal regulatory domains and a C-terminal kinase domain, and GTXX (W/Y) MAPE as Raf-specific signature was carried out by multiple alignment of kinase domains (Figs 1 and S1a). In contrast, a large number of ZIK members possessed an N-terminal kinase domain, and conserved signature motif GTPEFMAPE (L/V) (Y/F) was discovered in all members (Figs 2a and S1b). However, MEKK members possessed less conserved protein structures, with kinase domains that lay in either N- or C-terminal or the central part of the proteins and G (T/S) PX (F/Y/W) MAPEV forms a conserved signature of this group (Figs 2b and S1c), which is consistent with their orthologues in other plants^9,13^.

The pattern of amino acid residues found in many domains was evolutionarily conserved amongst MAPKKKs. Amongst the three groups, Raf MAPKKKs had the most members and greatest variety in primary structures and domain composition. Based on their evolutionary relationship and conserved motifs, we divided this groups into six subgroups (A–F), and the clades of each subgroup were numbered (Fig. S1a). The members of subgroup Raf-B contained a PB1 domain in the Fig. S1a, except for MdRaf2 and MdRaf15. The PB1 domain is required for formation of various complexes in different biological process^29^. Six subgroup-C Raf sequences had an aspartokinase, chorismate mutase, and TyrA (ACT) domain, which acted an important regulator of metabolic enzymes by sresponding to a wide range of amino acid concentrations^30^. Approximately 15 MAPKKKs in Raf-D had an EDR1 (enhanced disease resistance 1) domain (Fig. S1a). Previous work showed that in Arabidopsis, members of the Raf, CTR1 (constitutive triple response 1) and EDR1 acted as negative regulators in ethylene signalling and in the response to powdery mildew attack^26^. In addition, as shown in Fig. S1a, all members of Raf-F had an ANK (ankyrin repeat) domain in their N-terminal region, which was thought to function in protein recognition and protein-protein interaction^31^. MEKK was divided into four subgroups (I–IV) (Fig. S1c). MdMAPKKK4 in the MEKK IV subgroup had a TIR domain, which may suggest a further connection to plant defence-response signalling^26^.

Therefore, MAPKKKs members have similar gene structure and highly conserved motif among eukaryotes. It is speculated that most regions conserved amongst apple, maize, Arabidopsis and other species may be essential components that lay the foundation for gene functions. In contrast, other parts of the sequences may be formed in the process of gradual evolution, leading to differences in their functions.

### Chromosomal location and gene duplication of MdMAPKKKs

**Figure 3:**
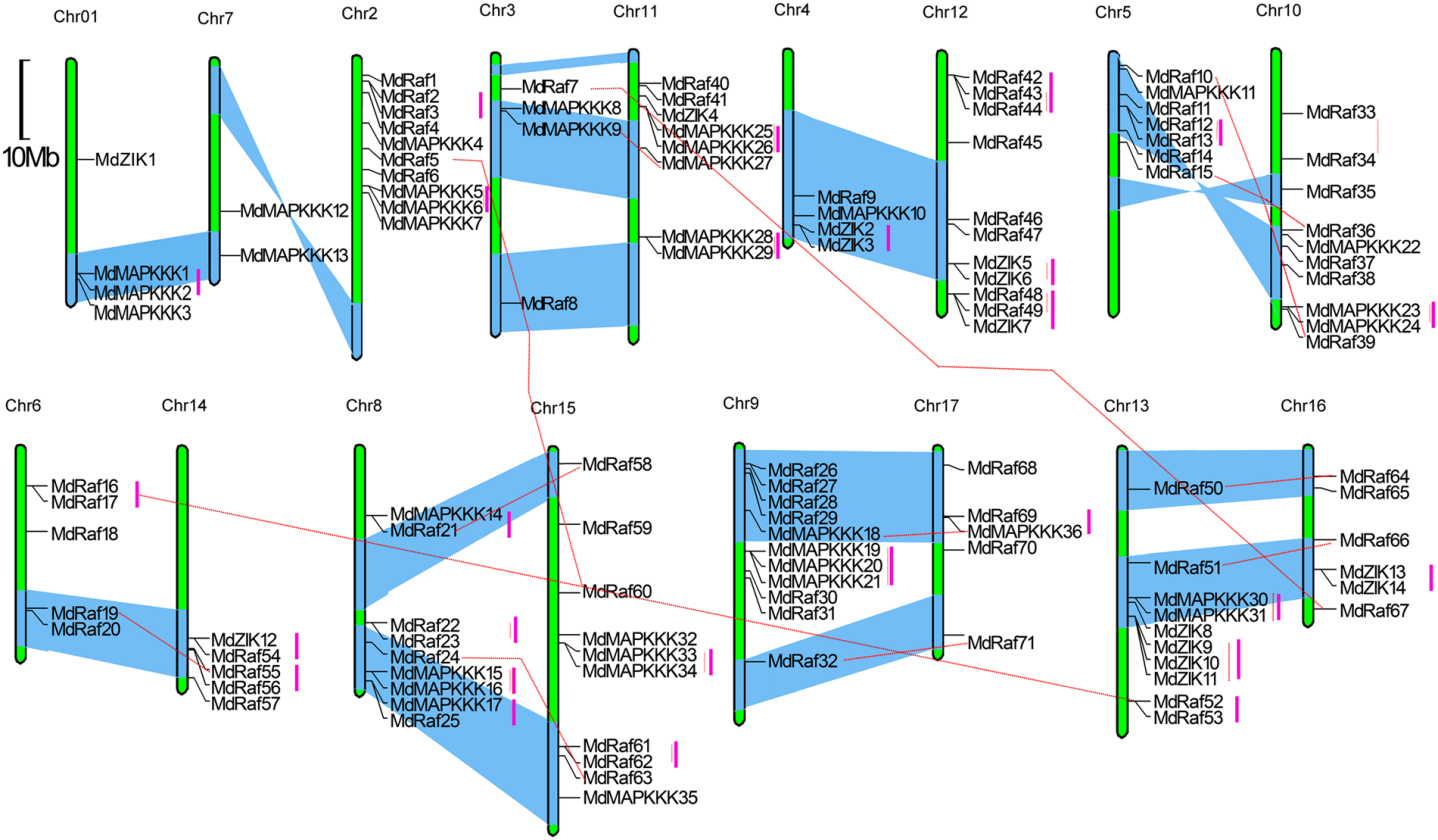
Positions of MAPKKK members on the apple chromosomes. The number of the chromosome is indicated on the top of each chromosome. Scale represents that the distance is 10 Mb. In order to simplify the presentation, we named the putative MAPKKK genes on the basis of the categories and the gene’s order on the chromosomes from Chr1 to Chr17. The segmented and homologous blocks that were duplicated are showed with a blue shadow. Sister paralogous pairs are showed by a red line.

Analysis of chromosomal location showed that 123 MdMAPKKK genes were mapped on all of 17 chromosomes in the apple with different densities. More than half were located on chromosomes 8 to 15 and dispersed throughout their respective genomes (Fig. 3). However two MAPKKKs (*MdMAPKKK37/MdRaf72*) were situated on unanchored contigs (chromosome unknown). Twenty-seven sister pair MdMAPKKKs and 4 clusters were tightly co-located in the apple genome, and 10 pairs were located in the segmental duplication area (Fig. 3). Gene duplications play a significant role in genomic rearrangement and expansion and have an important effect on diversification of gene function. It has been reported that a recent GWD event occurred in apple 60–65 million years ago, resulting in expansion of several gene classes that played vital roles in growth and development^25^. These include the Ring finger gene family^20^, LBD gene family^32^, DREB family^21^ and SBP gene family^23^. Therefore, we believe that duplications and transposition of chromosomal segments may have contributed to MdMAPKKKs expansion in apple.

### Synteny analysis of MdMAPKKKs between apple and Arabidopsis

**Figure 4:**
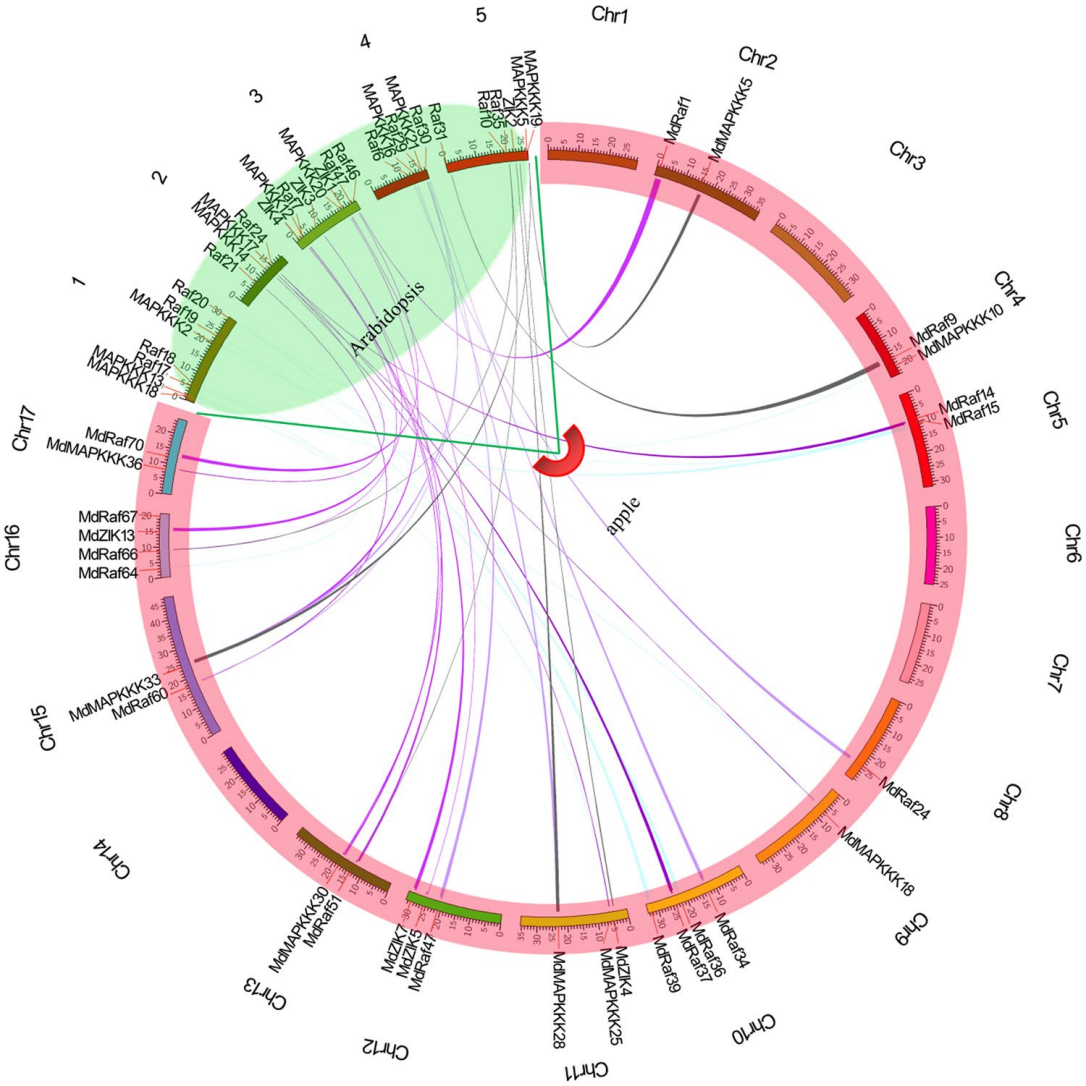
Synteny analysis of MAPKKK genes between apple and Arabidopsis. The positions of some related MdMAPKKK genes and AtMAPKKK genes are depicted in the apple’s and Arabidopsis’s chromosomes. The colored lines connect two chromosomal regions, which show the syntenic regions between the apple’s and the Arabidopsis’s chromosomes.

Genomic comparison is a quick and effective way to transfer genomic knowledge from one taxon, whose genomic structure, function and evolution are well understood, to a less-studied taxon^33^. Thus, MAPKKK genes’ probable function in apple can be deduced by comparison with orthologous genes in well-studied model plants Arabidopsis. In this study, synteny analysis for blocks duplicated between apple and Arabidopsis genomes showed that at least 38 pairs of MAPKKK genes from apple and Arabidopsis lay in syntenic genomic regions (Fig. 4). To elucidate the mechanisms of gene divergence after duplication of the MAPKKK genes in apple, the ratio of non-synonymous substitution rates (Ka) and synonymous substitution rates (Ks) was calculated. In this study, the values of Ka/Ks for the 38 duplicated orthologous gene pairs were all lower than 1 (Supplementary Table S3), which indicated that the MAPKKK genes from apple had mainly experienced purifying selection pressure after the segmental duplications as the previous study^34^. These results demonstrated that functions of the duplicated gene pairs in the MdMAPKKKs did not diverge as much from each other during subsequent evolution. This analysis provides an important foundation for the further functional dissection of the orthologous MAPKKKs in apple.

### Tissue-specific MdMAPKKKs’ expression pattern measured by qRT-PCR analysis

**Figure 5:**
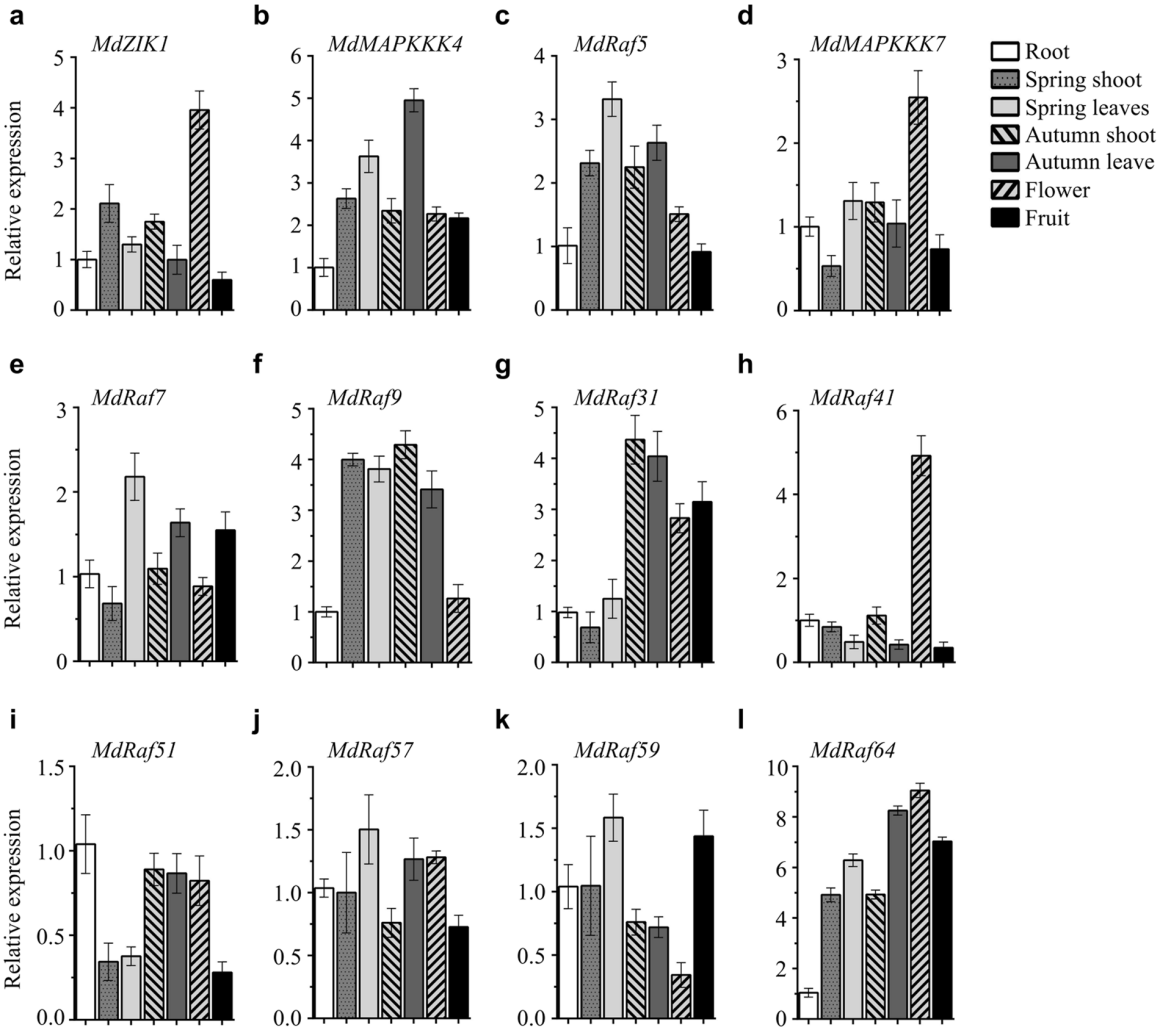
Tissue-specific expression profiles for the MdMAPKKK genes. The expression levels of MdMAPKKK genes (**a**) *ZIK1*, (**b**) *MdMAPKKK4*, (**c**) *MdRaf5*, (**d**) *MdMAPKKK7*, (**e)** *MdRaf7*, (**f**) *MdRaf9*, (**g**) *MdRaf31*, (**h**) *MdRaf41*, (**i**) *MdRaf51*, (**j**) *MdRaf57*, (**k**) *MdRaf59*, (**l**) *MdRaf64* were examined by qRT-PCR in roots, spring stems, spring leaves, autumn stems, autumn leaves, flowers and fruits in apple. The *18s rRNA* was performed as an internal control. Data are mean values of three biology repeats, and error bars indicate SD.

Increasing evidence has shown that MAPKKK genes are widely involved in growth and development during the lifecycle of the plant^26^. To gain insight into temporal and spatial transcription patterns and putative functions of MdMAPKKKs in apple growth and development, twelve MdMAPKKK genes (*ZIK1/MdMAPKKK4/MdRaf5/MdMAPKKK7/MdRaf7/MdRaf9/MdRaf31/MdRaf41/MdRaf51/MdRaf57/MdRaf59/MdRaf64*) were selected through a combined survey of categories and partial RNA-sequencing data (Supplementary Table S4) to confirm their expression using qRT-PCR in various tissues and stages, including the roots, spring shoots, spring leaves, autumn shoots, autumn leaves, flowers, and fruits. As shown in Fig. 5, most MdMAPKKKs exhibited a broad and constant expression pattern with transcripts detected in different developmental tissues. *MdRaf57* showed less expression in autumn shoots and fruits than in other tissues (Fig. 5j). *MdRaf59* and *MdRaf64*, exhibited high expression in all the above tissues, except for flowers and roots, respectively (Fig. 5k,l). *MdZIK1/MdMAPKKK7/MdRaf41* exhibited similar higher transcript abundance in flowers but lower levels in other tissues, even though they were from various groups, respectively (Fig. 5a,d,h). In addition, expression levels of *MdRaf31/MdRaf51* in autumn shoots, autumn leaves, and flowers were higher than those in spring shoots and spring leaves (Fig. 5g,i). *MdRaf5/MdRaf9* in the same subgroup were highly expressed in most tissues, but they displayed very low expression in roots and fruits (Fig. 5c,f). This findings suggested that the time when shoot growth occurs during the growing season might influence induction of certain MdMAPKKKs and their transcripts abundance. Most MdMAPKKKs in the same subgroup share a highly conserved structure and similar expression patterns, suggesting the potential intimate evolution relationship and functional redundancy exist between the duplicated genes. To extract information about the relative abundance of transcripts of Arabidopsis and *O*. *sativa* MAPKKKs, we conducted analyses in genevestigator (https://genevestigator.com/gv/). We found 73 genes at 10 developmental stages of Arabidopsis and 71 genes at 9 developmental stages in rice that exhibited different expression levels in many types of organs and tissues (Figs S4–S7). Therefore, MdMAPKKKs may function principally in organ- or tissue-specific development in apple as other plants.

### Expression profiles of MAPKKK genes under drought in different species

MAPKKKs have been reported to play a vital role in various stresses tolerance in various species, including Arabidopsis, cotton, rice, and maize^35–38^. Amongst these stressors, drought is a major environmental factor limiting productivity and distribution of plants^39^. Previous studies have reported that MAPKKK members DSM1 (Drought Hypersensitive Mutant 1) and MAPKKK18 mediate drought stress tolerance^37,40^. However, no one MdMAPKKKs correlated to drought stress resistance is elucidated in apple. To identify effects of MdMAPKKK genes expression on drought responses, apple seedlings were treated with 20% PEG for 3 h. We examined expression levels of 12 MdMAPKKKs in roots and leaves using qRT-PCR.

**Figure 6:**
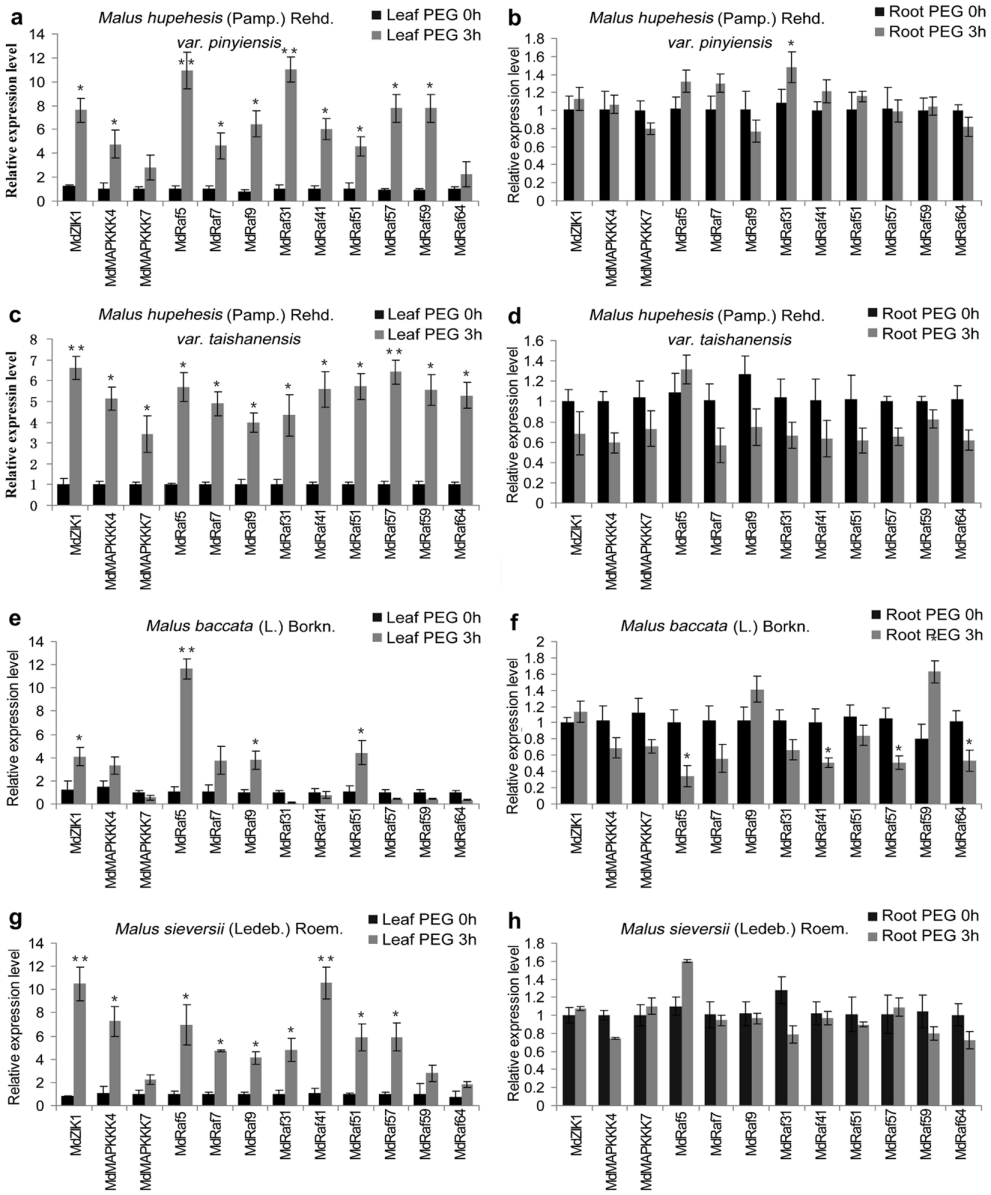
The expression profiles of the identified MdMAPKKKs in roots and leaves of four species under drought conditions by qRT-PCR analysis. (**a**) Expression profiles of the identified MdMAPKKKs in the leaves of *Malus hupehesis* (Pamp.) Rehd. *var*. *pinyiensis*, (**b**) Expression profiles of the identified MdMAPKKKs in the roots of *Malus hupehesis* (Pamp.) Rehd. *var*. *pinyiensis*, (**c**) Expression profiles of the identified MdMAPKKKs in the leaves of *Malus hupehesis* (Pamp.) Rehd. *var*. *taishanensis*, (**d**) Expression profiles of the identified MdMAPKKKs in the roots of *Malus hupehesis* (Pamp.) Rehd. *var*. *taishanensis*, (**e**) Expression profiles of the identified MdMAPKKKs in the leaves of *Malus baccata* (L.) Borkn, (**f**) Expression profiles of the identified MdMAPKKKs in the roots of *Malus baccata* (*L*.) Borkn, (**g**) Expression profiles of the identified MdMAPKKKs in the leaves of *Malus sieversii* (Ledeb.) Roem, (**h**) Expression profiles of the identified MdMAPKKKs in the roots of *Malus sieversii* (Ledeb.) Roem. Data are mean values of three biology repeats, and error bars indicate SD. Statistical significance between the PEG 0h and PEG 3h were determined by Student's *t*-test: *P < 0.05; **P < 0.01; ***P < 0.001.

Expression patterns showed very interesting results: in all four apple species, all 12 MAPKKKs were highly regulated by PEG in leaves but only slightly induced in roots (Fig. 6). In *Malus hupehesis* (Pamp.) Rehd. *var*. *pinyiensis*, all 12 MAPKKKs were up-regulated at least 2-fold in leaves. *MdRaf5* and *MdRaf31* exhibited the highest up-regulation, reaching approximately 10-fold in 3 h (Fig. 6a). In *Malus hupehesis* (Pamp.) Rehd. *var*. *taishanensis*, all genes were dramatically up-regulated 3- to 7-fold in leaves (Fig. 6c). In *Malus baccata* (L.) Borkn, six of MAPKKK genes (*MdZIK1/MdMAPKKK4/MdRaf5/MdRaf7/MdRaf9/MdRaf51*) showed up-regulated transcript levels, whilst other genes were reduced in leaves (Fig. 6e). In *Malus sieversii* (Ledeb.) Roem, all 12 MAPKKK genes were significantly up-regulated by PEG in leaves, and there was no significant change in roots (Fig. 6g,h). These findings indicated a feasible role for MdMAPKKKs in drought resistance in leaves and expanded our understanding of corresponding molecular mechanisms regulated by MAPK cascades in modulating abiotic stress responses in apple.

### Overexpression of *MdRaf5* enhances the drought stress tolerance in transgenic Arabidopsis

**Figure 7:**
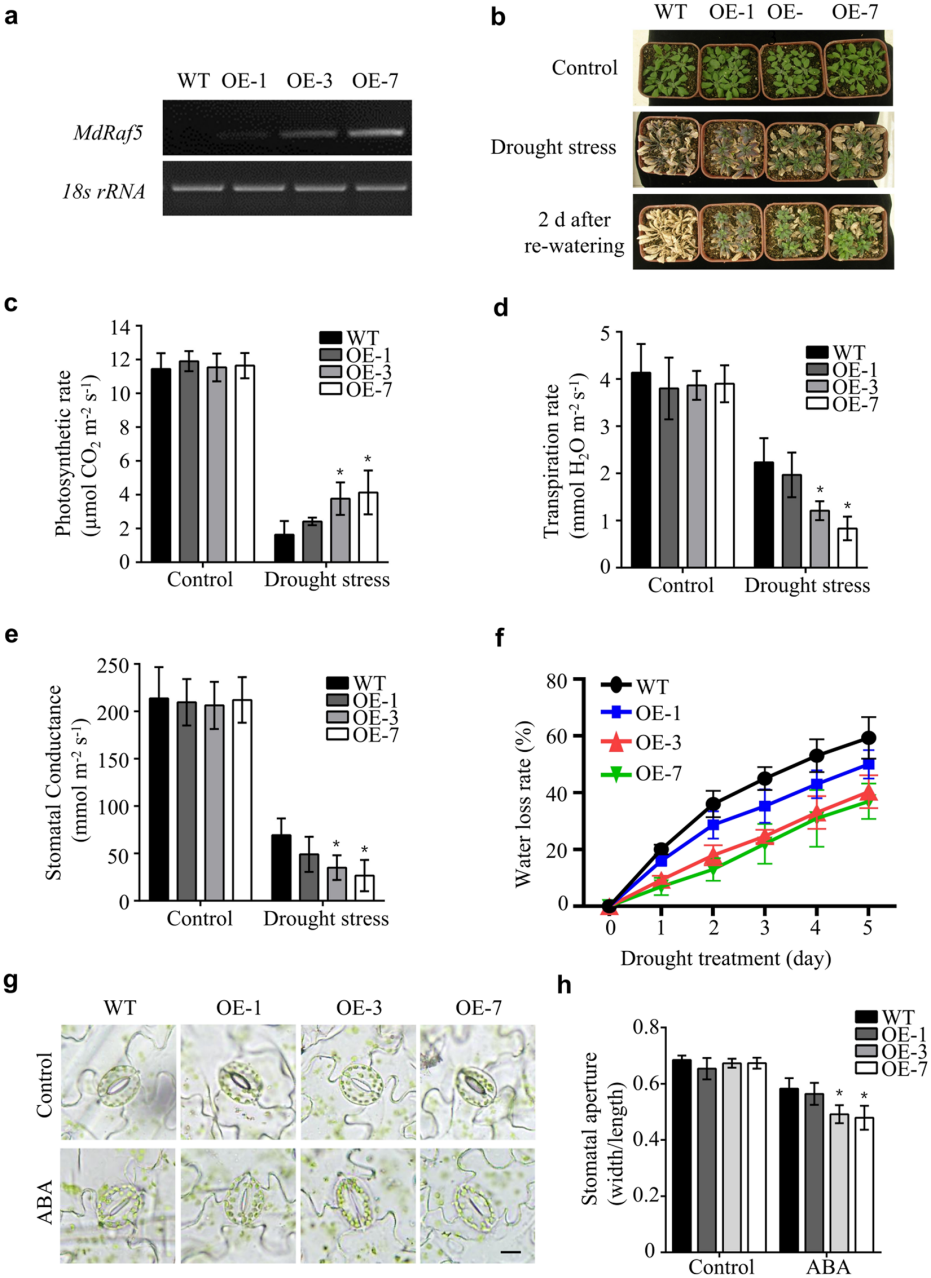
*35S*::MdRaf5 transgenic plants show improved resistance to drought-stress in Arabidopsis. (**a**) Semi-quantitative PCR showed that *MdRaf5* was overexpressed and three lines (OE-1, -3, -7) were chosen for drought stress treatment. (**b**) Drought-resistant phenotype of overexpressing *MdRaf5* in Arabidopsis. 30 d old Arabidopsis plants was withheld from water for 20 d and re-watered for 2 d. (**c**) Net photosynthetic rate, (**d**) Transpiration rates, (**e**) Stomatal conductance, (**f**) Water loss measured with or without drought stress treatment, (**g**) Stomatal changes observed with a microscope with or without ABA treatment, (**h**) Relative stomatal aperture from leaves of the WT (Col-0) and *35S*::*MdRaf5* transgenic Arabidopsis with or without ABA stress. Data are mean values of three biology repeats, and error bars indicate SD. Statistical significance between the WT and the overexpression lines were determined by Student's *t*-test: *P < 0.05; **P < 0.01.

*MdRaf5*, a function unknown Group C Raf-like MAPKKK, was significantly induced by PEG treatment in all four apple species, prompting us to investigate whether the Group C Raf-like MAPKKK member participated in drought stress. *MdRaf5* overexpression transgenic Arabidopsis plants were generated and three overexpression lines (OE1, OE3 and OE7) were used for drought tolerance assays (Fig. 7a). As shown in Fig. 7b, almost no wild type plants survived with drought treatment, which consisted of withholding water for 20 d. In contrast, most transgenic plants recovered after re-watering, suggesting that overaccumulation of *MdRaf5* unambiguously enhanced drought stress tolerance in transgenic Arabidopsis.

Reduction of transpirational water loss is one of the main factors that contribute to drought tolerance. We evaluated specific effects of drought stress on photosynthetic gas exchange parameters between different genotypes in our study. As shown in Fig. 7c–e, there were no significant differences in photosynthetic gas exchange parameters between transgenic and WT plants under standard conditions. Drought stress resulted in a marked decrease in all parameters in both transgenic and WT plants. The net photosynthesis rates was maintained at a higher level in OE lines than in WT under drought stress, whereas OE lines had dramatically lower transpiration rates and stomatal conductance than WT plants (Fig. 7c–e). To measure the rate of water loss, fresh weight changes in aerial parts from WT and OE lines were recorded over 5 h. Consistent with drought stress assay results, OE line leaves showed much slower water loss than WT leaves (Fig. 7f). In addition, we observed that the OE lines displayed faster ABA-induced stomatal closure than that of WT to reduce transpiration rates and water loss (Fig. 7g,h). We speculate that different transpiration rates, water loss and stomatal aperture in leaves are likely to be one of the main reasons for divergent drought stress tolerance observed in WT and OE plants. Therefore, MdRaf5 likely exerted a regulatory role by accelerating stomatal closure and decreasing transpiration rates when drought stress occurred.

## Discussion

Plants possess integrated signalling networks that mediate responses to environmental stressors and transmit extracellular signals to their intracellular targets. MAPKs are an important, evolutionarily conserved signal transduction cascade that connects diverse membrane receptors/sensors to a wide range of cellular responses in mammals, yeasts and plants, including various stress responses, hormone responses, cell division, and PAMP-triggered immunity^5,41–44^.

A MAPK cascade is composed of three kinases, MAPK, MAPKK, and MAPKKK^14,45^. The three kinases form the basic module of a MAPK cascade, MAPKKK-MAPKK-MAPK, which is activated via phosphorylation. MAPKKKs are serine/threonine kinases that can phosphorylate two amino acids in the S/T-X_3_-_5_-S/T motif of MAPKKs; MAPKKs are dual-specificity kinases that activate a MAPK by double phosphorylation of the T-X-Y motif in the activation loop^26^; and MAPKs are serine/threonine kinases that can phosphorylate various cytoplasmic or nuclear substrates, including other kinases and transcription factors^46^.

The MAPKKKs have more members than MAPK and MAPKK, and these members can be subdivided into MEKK, ZIK-like and Raf-like groups^15^. As important components of MAPK signal transduction modules, MAPKKKs have a significant regulatory effect on several biological processes in plants, such as the response to various stressors, plant cytokinesis, ethylene signalling, innate immunity and defence responses^4^. Amongst these three groups, the MEKK has been relatively well characterized. The *NPK1* (*Nicotiana tabacum* protein kinase 1) gene in tobacco may lead to cytokinesis by interacting with its activators and proteins such as kinesin^47^. *OMTK1* (*oxidative stress-activated MAP triple-kinase 1*), a MAPKKK gene from *M*. *sativa*, has been found to participate in oxidative stress signalling^48^. Further ANPs in Arabidopsis are shown to be participated in the oxidative-stress-response and act as negative regulators of the auxin-response pathway^26,49^. *YODA*, a MAPKKK in Arabidopsis is involved in stomatal development by targeting downstream MAPKKs^44,50^. In addition, the MAPK signalling cascade (MEKK1-MKK4/5-MPK3/6-FRK1/WRKY29) plays a decisive role in innate immunity of plants^41^. Moreover, stress-induced MEKK1 can activate MKK2 and its subsequent targets MPK4 and MPK6. This pathway has been functionally implicated in signalling pathway related to oxidative, salt, and cold stresses as well as in innate plant immunity^51^. In rice, *OsMAPKKK28* and *OsMAPKKK8*, which belongs to the MEKK, are involved in drought stress, whereas *OsMAPKKK63* is involved in cold stress^9^. Recently, a member of the ZIK in Arabidopsis, WNK1 (At3g04910), has been implicated in control of flowering time and circadian rhythms^52^. Raf MAPKKKs have the most members amongst the three groups. However, only a small fraction of Raf-like MAPKKKs have been identified and studied at the molecular and genetic levels to characterize their biological functions, including Arabidopsis CTR1, EDR1, SIS8 (sugar insensitive 8) and MAP3Kδ4 as well as rice ERD1 and DSM1. CTR1 and EDR1 act as negative regulators in ethylene signalling and respond to salicylic acid (SA)-inducible powdery mildew attack, respectively in Arabidopsis^53^. SIS8 is involved in salt stress and sugar responses, whereas MAP3Kδ4 has been shown to regulate growth and shoot branching^38,54^. Nevertheless, the detailed biological functions of most MAPKKKs, especially Raf MAPKKKs are unclear at present.

In this study, we first identified 123 MAPKKKs that contained full ORFs in apple using genome-wide analysis (Supplementary Table S1). The number of MAPKKKs identified in apple was approximately 1.5-fold higher than that discovered in Arabidopsis, rice and maize, which indicated that MAPKKKs had expanded in apple. Chromosomal location analyses indicated that the MdMAPKKK genes mapped to all 17 chromosomes in apple, and there were expected gene duplications amongst those chromosomes (Fig. 3). Twenty-seven sister pair MdMAPKKKs and 4 clusters were tightly co-located in the apple genome, and 10 pairs were located in the segmental duplication area (Fig. 3). Gene duplication plays a significant role in the genomic rearrangement and expansion, which is the main contributor to evolutionary momentum^25^. Apple have the largest MAPKKKs amongst the species mentioned above, suggesting that there are more complex evolutionary relationships amongst MAPKKKs in apple, and more genes are needed for complicated transcriptional regulations in this woody perennial plant. Thus, the whole genome as well as duplications and transposition of chromosomal segments may have contributed to apple MdMAPKKKs expansion in evolution.

Phylogenetic analysis based upon the unrooted tree of MAPKKKs from apple and Arabidopsis has allowed us to first classify these apple genes into MEKK, ZIK-like and Raf-like groups first and then further categorize each subgroup (Figs S1 and S2). In addition, detailed information regarding apple MAPKKK genes, including genomic position, ORF length, numbers of amino acids, molecular weight, and isoelectric points (pIs) were also predicted using web resources (Supplementary Table S1). The most closely linked members in the same subgroups shared a similar gene structure and conserved domain in apple, which suggested that they might have closer phylogenetic relationships and similar functions, and the further studies needed to carry out. Results of qRT-PCR showed that MAPKKKs were maximally expressed in all tissues (Fig. 5), but some exhibited variable expression levels in different tissues, suggesting functional divergence amongst MAPKKKs during plant growth and development. Drought stress treatments indicated that MdMAPKKKs played important roles in regulating drought stress signalling pathways (Fig. 6). MdRaf5, a biological function unknown Group C Raf-like MAPKKK, dramatically induced by drought treatment in four various species (Fig. 6). Overexpression of MdRaf5 in transgenic Arabidopsis dramatically enhanced drought tolerance through reducing transpiration rates and stomatal aperture, and the current study was the first investigation of its role (Fig. 7).

To the best of our knowledge, large numbers of MAPKKKs have been implicated in drought stress in previous studies. *DSM1*, a Group B Raf-like MAPKKK in rice, and *GhRaf19*, a Group C Raf-like MAPKKK in cotton, positively and negatively regulate resistance to drought stress through reactive oxygen species scavenging, respectively^35,37^. Libraries made from rice roots under salinity and cold stress have shown maximum transcript abundance of *OsMAPKKK64*, a member of the ZIK, which is elevated under drought stress^9^. In addition, *OsMAPKKK28* and *OsMAPKKK8* have been implicated in drought stress through unknown pathways^9^. The MAPK signalling cascade AtMEKK1-AtMKK2-AtMPK4/AtMPK6 may activate RD29A through expression of DREB1 protein to increase abiotic stress including drought stress in plants^55^. YODA-MKK4/MKK5-MPK3/MPK6 cascade is as a key component of intercellular interactions to control stomatal development to respond to drought stress^50,56^. Although significant progress has been made in discerning how plants respond to water shortage, drought stress resistance associated canonical MAPKKKs have not been established in apple. In our study, the Raf C-like *MdRaf5* overexpression phenotype demonstrated the positive role of MdRaf5 in responding to drought stress by regulating stomatal aperture and transpiration rate (Fig. 7). Therefore, it is possible that MdRaf5 mainly exerted its regulatory roles via downstream MAPKK and MAPK to modulate stomatal closure and drought stress as YODA does. Further screening of proteins interacting with MdRaf5 will provide new information regarding mechanisms for the function of MdRaf5 in abiotic stress responses. Overall, the comprehensive analysis of MdMAPKKKs in the present study will serve the purpose of illuminating poorly characterized MAPK kinase cascades in apple. Additionally, our study has laid a solid foundation for further functional investigation into the molecular mechanisms of MAPKKKs and, ultimately, MAPK kinase cascades in response to extracellular stimuli as well as their central roles in various biological processes in apple.

## Materials and Methods

### Identification of MdMAPKKKs in apple

Searches of multiple databases were performed in order to identify members of the MAPKKKs. MAPKKKs sequences for Arabidopsis and rice were used as queries to perform repetitive blast searches against the GDR database (Genome Database for *Rosaceae*: http://www.rosaceae.org/). Additionally, all protein sequences of proteins were then used as queries to perform multiple database searches against proteome and genome files downloaded from GDR. Stand-alone versions of BLASTP (Basic Local Alignment Search Tool: http://blast.ncbi.nlm.nih.gov) and TBLASTN available from NCBI (National Center for Biotechnology Information) were used with an e-value cutoff of 1 × 10^−355^. Furthermore, predicted sequences for the MAPKKKs were downloaded in the Apple GFDB database (Apple Gene Function and Gene Family Database: http://www.applegene.org/). All protein sequences derived from the candidate MAPKKK genes were collected and examined with the domain analysis programs Pfam (Protein family: http://pfam.sanger.ac.uk/) and SMART (Simple Modular Architecture Research Tool: http://smart.embl-heidelberg.de/) with default cutoff parameters^57^. With the help of proteomics and sequence analysis tools available on the ExPASy Proteomics Server (http://expasy.org/), isoelectric points and molecular weights of MdMAPKKKs were obtained^58^.

### The Chromosomal location and structure of MdMAPKKK genes

Chromosomal locations and gene structures retrieved from apple genome data were downloaded from the GDR database. Other genes were sampled and selected using a Perl-based program. Chromosomes were drawn with MapDraw^59^, and MdMAPKKKs structures were generated with GSDS (Gene Structure Display Server: http://gsds.cbi.pku.edu.cn/).

### Synteny analysis of MdMAPKKKs between apple and Arabidopsis

Synteny blocks of the apple and Arabidopsis genomes were downloaded from the Plant Genome Duplication Database (http://chibba.agtec.uga.edu/duplication), and those containing apple MAPKKK genes were additionally measured and analysed. The diagram was finished with Circos^60^.

### Sequence alignment and phylogenetic analysis

MdMAPKKK sequences were aligned with the program ClustalX using BLOSUM30 as the protein weight matrix. The MUSCLE (Multiple Sequence Comparison by Log-Expectation) program (version 3.52) was additionally used to perform multiple sequence alignments to confirm ClustalX data output (http://www.clustal.org/). Phylogenetic trees based on MdMAPKKK protein sequences were constructed with the NJ method of the program MEGA5 with p-distance and complete deletion option parameters engaged. Reliability of the derived trees was tested using bootstrapping with 1,000 replicates. Phylogenetic tree images were drawn with MEGA5.

### Plant’s growth, treatments and tissues collection

Different tissues (root, spring stem, spring leaf, autumn stem, autumn leaf, flower and fruit) of *Malus hupehensis* (*Malus hupehesis* (Pamp.) Rehd. *var*. *pinyiensis*, the distinguished apple rootstock widely used for grafting in China) were used to quantify tissue-specific expression patterns of MAPKKK genes in apple. *Malus hupehensis* trees were 12 years old and had been planted in the Experimental Orchard of Shandong Institute of Fruit Tree Science (Tai’an, China).

We selected four wild varieties for drought stress testing, including *Malus hupehesis* (Pamp.) Rehd. *var*. *pinyiensis*, *Malus hupehesis* (Pamp.) Rehd. *var*. *taishanensis*, *Malus baccata* (L.) Borkn, and *Malus sieversii* (Ledeb.) Roem. Seeds were stratified at 4 °C for 40 d, and the seedlings were transplanted into 50% Hoagland’s nutrient solution under greenhouse conditions under 16/8 h light/dark cycles at 26±1 °C. After 30 d, uniformly developed seedlings at the three- or four-leaf stage were selected for stress treatments. For drought treatments, seedlings were cultured in 50% Hoagland’s nutrient solution containing 20% PEG 6000 for 0 and 3 h. Then, roots and leaves were collected, rapidly frozen in liquid nitrogen, and stored at −80 °C. Each treatment was repeated three times.

### RNA extraction, cDNA synthesis and qRT-PCR analysis

Total RNA was extracted using Plant RNA Purification Reagent (Invitrogen, CA, USA) as described in previous research report and was treated with RNase-free DNase I (Invitrogen, USA)^61^. First-strand cDNA was synthesized using two micrograms of total RNA with PrimeScript First Strand cDNA Synthesis Kit (Takara, China). MdMAPKKK genes transcript levels were measured by qRT-PCR. qRT-PCR was performed using gene-specific primers and SYBR Green master mix (Vazyme Biotech co.,ltd) on a Bio-Rad iCycler. The fold changes in gene expression were calculated by the 2^−ΔΔCt^ comparative CT method. The 18s ribosomal RNA genes were used as internal normalisation controls. Each test of gene expression was three replicates. Supplementary Table S5 lists relevant details of the primers.

### Gene cloning and genetic transformation

ORFs of *MdRaf5* were amplified from *Malus hupehesis* (Pamp.) Rehd. *var*. *pinyiensis* cDNA using primer 5′-GAATCCATGGCTATCGACGAGGACGTGG and 3′-GTCGACTCAGTGATTGCCCCATCTAAGT, and cloned into the binary vector pBI121 under control of the original CaMV35S promoter. All plasmid linkages were confirmed by sequencing. The pBI121-MdRaf5 plasmid was transformed into Arabidopsis ecotype Col-0 using *Agrobacterium tumefaciens* GV3101. All transgenic plants were screened for kanamycin and hygromycin resistance and verified by PCR. T_2_ generation plants were used for the drought stress resistance test. Arabidopsis seeds were surface-sterilized with 20% bleach for 10 minutes and rinsed four times in sterile deionized water. The sterilized seeds were grown vertically in 0.3% Phytagel medium containing 50% Murashige and Skoog nutrients (PhytoTech) and 1% sucrose (pH = 5.7) and kept at 4 °C for 3 d. For drought stress analysis, Arabidopsis plants were grown in a growth room at 22 °C under a 16/8 h light/dark cycle.

### Drought-Stress Treatments for the transgenic Arabidopsis

The T_2_ generation of transgenic Arabidopsis was subjected to the drought stress resistance test in soil. All seeds were imbibed at 4 °C for 3 d and tiled in 50% Murashige and Skoog solid medium. Five days after germination, seedlings were directly planted in soil. Drought treatment was imposed for 20 d beginning 30 d after plants emerged. Plants were re-watered on day 51 (1 d after the 20 d drought treatment) and assessed for survival 2 d later.

### Measurement of net photosynthesis rates, stomatal conductance, transpiration rates, water Loss and stomatal aperture

Thirty-day-old plants from the WT and OE lines were held with or without water for 10 d before the plant withered and died, and net photosynthetic rate, stomatal conductance and transpiration rate were subsequently measured using a portable photosynthetic system (CIRAS-3, PP Systems, Hitchin, UK) in the morning between 9:00–11:00. Photosynthesis was measured indoors. CO_2_ concentration was controlled at 380 ppm. Light intensity was controlled at 1000 μmolm^−2^ s^−1^ PPFD, and relative humidity ranged from 60–70%. The temperature inside the leaf chamber was 25 °C. In another experiment, aboveground parts of the 30-d-old plants which were grown under normal conditions were collected and weighed before and after drying. For measurement of water loss, 30-d-old plants’ rosettes were cut off from the base and weighed at the scheduled time.

Mature leaves from 3-week-old plants were exposed to light for 3 h after incubation in stomatal opening solution (1 M MES, 1 M KCl, 100 mM CaCl_2_, H_2_O, pH 6.2) in the dark for 30 min. Then, the opening solution was supplemented with ABA or not (final concentration 5 mM) and the stomatal aperture was observed at 2 h after ABA treatment. Abaxial epidermal peels were peeled by the tweezers and then the epidermis was placed on a slide. Stomatal aperture was analyzed under a confocal laser scanning microscope (LSM710; Carl Zeiss AG, Oberkochen, Germany). Ten photos were taken for each epidermal peel, and used for measuring stomatal aperture with the micrometer. The stomatal aperture was calculated by the mean ratio of width to length of 20 stomatal apertures. This experiment was repeated three times.

## Electronic supplementary material

Supplementary information

## References

[1] Nolen B, Taylor S, Ghosh G (2004). Regulation of protein kinases; controlling activity through activation segment conformation. Molecular cell.

[2] Tena G, Boudsocq M, Sheen J (2011). Protein kinase signaling networks in plant innate immunity. Current opinion in plant biology.

[3] Zulawski M, Schulze G, Braginets R, Hartmann S, Schulze WX (2014). The Arabidopsis Kinome: phylogeny and evolutionary insights into functional diversification. BMC genomics.

[4] Xu J, Zhang S (2015). Mitogen-activated protein kinase cascades in signaling plant growth and development. Trends in plant science.

[5] Cardinale F, Meskiene I, Ouaked F, Hirt H (2002). Convergence and divergence of stress-induced mitogen-activated protein kinase signaling pathways at the level of two distinct mitogen-activated protein kinase kinases. The Plant cell.

[6] Colcombet J, Hirt H (2008). Arabidopsis MAPKs: a complex signalling network involved in multiple biological processes. The Biochemical journal.

[7] Jonak C, Okresz L, Bogre L, Hirt H (2002). Complexity, cross talk and integration of plant MAP kinase signalling. Current opinion in plant biology.

[8] Sinha AK, Jaggi M, Raghuram B, Tuteja N (2011). Mitogen-activated protein kinase signaling in plants under abiotic stress. Plant signaling & behavior.

[9] Rao KP, Richa T, Kumar K, Raghuram B, Sinha AK (2010). In silico analysis reveals 75 members of mitogen-activated protein kinase kinase kinase gene family in rice. DNA research: an international journal for rapid publication of reports on genes and genomes.

[10] Kong X (2013). Genome-wide identification and analysis of expression profiles of maize mitogen-activated protein kinase kinase kinase. PloS one.

[11] Wu J (2014). Genome-wide identification of MAPKK and MAPKKK gene families in tomato and transcriptional profiling analysis during development and stress response. PloS one.

[12] Zhang X (2016). Integration analysis of MKK and MAPK family members highlights potential MAPK signaling modules in cotton. Scientific reports.

[13] Wang J (2015). Genome-wide identification of MAPK, MAPKK, and MAPKKK gene families and transcriptional profiling analysis during development and stress response in cucumber. BMC genomics.

[14] Davis S, Laroche S (2006). Mitogen-activated protein kinase/extracellular regulated kinase signalling and memory stabilization: a review. Genes, brain, and behavior.

[15] Hamel LP, Sheen J, Seguin A (2014). Ancient signals: comparative genomics of green plant CDPKs. Trends in plant science.

[16] Wrzaczek M, Hirt H (2001). Plant MAP kinase pathways: how many and what for?. Biology of the cell.

[17] Wang G (2014). Genome-wide identification and analysis of mitogen activated protein kinase kinase kinase gene family in grapevine (Vitis vinifera). BMC plant biology.

[18] Popescu SC (2009). MAPK target networks in Arabidopsis thaliana revealed using functional protein microarrays. Genes & development.

[19] Lee YP (2007). Microarray analysis of apple gene expression engaged in early fruit development. Plant cell reports.

[20] Li Y (2011). Genome-wide analysis of the RING finger gene family in apple. Mol Genet Genomics.

[21] Zhao T, Liang D, Wang P, Liu J, Ma F (2012). Genome-wide analysis and expression profiling of the DREB transcription factor gene family in Malus under abiotic stress. Mol Genet Genomics.

[22] Liang D, Xia H, Wu S, Ma F (2012). Genome-wide identification and expression profiling of dehydrin gene family in Malus domestica. Mol Biol Rep.

[23] Giorno F, Guerriero G, Baric S, Mariani C (2012). Heat shock transcriptional factors in Malus domestica: identification, classification and expression analysis. BMC genomics.

[24] Zhang S, Xu R, Luo X, Jiang Z, Shu H (2013). Genome-wide identification and expression analysis of MAPK and MAPKK gene family in Malus domestica. Gene.

[25] Velasco R (2010). The genome of the domesticated apple (Malus x domestica Borkh.). Nature genetics.

[26] Group M (2002). Mitogen-activated protein kinase cascades in plants: a new nomenclature. Trends in plant science.

[27] Lehti-Shiu MD, Shiu SH (2012). Diversity, classification and function of the plant protein kinase superfamily. Philosophical transactions of the Royal Society of London. Series B, Biological sciences.

[28] Wu J (2013). The genome of the pear (Pyrus bretschneideri Rehd.). Genome research.

[29] Sumimoto H, Kamakura S, Ito T (2007). Structure and function of the PB1 domain, a protein interaction module conserved in animals, fungi, amoebas, and plants. Science’s STKE: signal transduction knowledge environment.

[30] Aravind L, Koonin EV (1999). Gleaning non-trivial structural, functional and evolutionary information about proteins by iterative database searches. Journal of molecular biology.

[31] Gorina S, Pavletich NP (1996). Structure of the p53 tumor suppressor bound to the ankyrin and SH3 domains of 53BP2. Science.

[32] Wang X, Zhang S, Su L, Liu X, Hao Y (2013). A genome-wide analysis of the LBD (LATERAL ORGAN BOUNDARIES domain) gene family in Malus domestica with a functional characterization of MdLBD11. PloS one.

[33] Lyons E (2008). Finding and comparing syntenic regions among Arabidopsis and the outgroups papaya, poplar, and grape: CoGe with rosids. Plant physiology.

[34] Liu W (2014). Genome-wide survey and expression analysis of calcium-dependent protein kinase in Gossypium raimondii. PloS one.

[35] Jia H (2016). A Raf-like MAPKKK gene, GhRaf19, negatively regulates tolerance to drought and salt and positively regulates resistance to cold stress by modulating reactive oxygen species in cotton. Plant science: an international journal of experimental plant biology.

[36] Liu Y (2015). RNA-Seq Analysis Reveals MAPKKK Family Members Related to Drought Tolerance in Maize. PloS one.

[37] Ning J, Li X, Hicks LM, Xiong L (2010). A Raf-like MAPKKK gene DSM1 mediates drought resistance through reactive oxygen species scavenging in rice. Plant physiology.

[38] Virk N (2015). Arabidopsis Raf-Like Mitogen-Activated Protein Kinase Kinase Kinase Gene Raf43 Is Required for Tolerance to Multiple Abiotic Stresses. PloS one.

[39] Boyer JS (1982). Plant productivity and environment. Science.

[40] Li Y (2017). Arabidopsis MAPKKK18 positively regulates drought stress resistance via downstream MAPKK3. Biochemical and biophysical research communications.

[41] Asai T (2002). MAP kinase signalling cascade in Arabidopsis innate immunity. Nature.

[42] Singh R, Jwa NS (2013). The rice MAPKK-MAPK interactome: the biological significance of MAPK components in hormone signal transduction. Plant cell reports.

[43] Gustin MC, Albertyn J, Alexander M, Davenport K (1998). MAP kinase pathways in the yeast Saccharomyces cerevisiae. Microbiology and molecular biology reviews: MMBR.

[44] Zhang T (2006). Diverse signals converge at MAPK cascades in plant. Plant physiology and biochemistry: PPB.

[45] Lee LK (2014). A Review of Signal Transduction of Endothelin-1 and Mitogen-activated Protein Kinase-related Pain for Nanophysiotherapy. Journal of physical therapy science.

[46] Fiil BK, Petersen K, Petersen M, Mundy J (2009). Gene regulation by MAP kinase cascades. Current opinion in plant biology.

[47] Ishikawa M, Soyano T, Nishihama R, Machida Y (2002). The NPK1 mitogen-activated protein kinase kinase kinase contains a functional nuclear localization signal at the binding site for the NACK1 kinesin-like protein. The Plant journal: for cell and molecular biology.

[48] Nakagami H, Kiegerl S, Hirt H (2004). OMTK1, a novel MAPKKK, channels oxidative stress signaling through direct MAPK interaction. The Journal of biological chemistry.

[49] Kovtun Y, Chiu WL, Zeng W, Sheen J (1998). Suppression of auxin signal transduction by a MAPK cascade in higher plants. Nature.

[50] Wang H, Ngwenyama N, Liu Y, Walker JC, Zhang S (2007). Stomatal development and patterning are regulated by environmentally responsive mitogen-activated protein kinases in Arabidopsis. The Plant cell.

[51] Teige M (2004). The MKK2 pathway mediates cold and salt stress signaling in Arabidopsis. Molecular cell.

[52] Murakami-Kojima M, Nakamichi N, Yamashino T, Mizuno T (2002). The APRR3 component of the clock-associated APRR1/TOC1 quintet is phosphorylated by a novel protein kinase belonging to the WNK family, the gene for which is also transcribed rhythmically in Arabidopsis thaliana. Plant & cell physiology.

[53] Clark KL, Larsen PB, Wang X, Chang C (1998). Association of the Arabidopsis CTR1 Raf-like kinase with the ETR1 and ERS ethylene receptors. Proc Natl Acad Sci USA.

[54] Huang Y, Li CY, Qi Y, Park S, Gibson SI (2014). SIS8, a putative mitogen-activated protein kinase kinase kinase, regulates sugar-resistant seedling development in Arabidopsis. The Plant journal: for cell and molecular biology.

[55] Hua ZM, Yang X, Fromm ME (2006). Activation of the NaCl- and drought-induced RD29A and RD29B promoters by constitutively active Arabidopsis MAPKK or MAPK proteins. Plant, cell & environment.

[56] Bergmann DC, Lukowitz W, Somerville CR (2004). Stomatal development and pattern controlled by a MAPKK kinase. Science.

[57] Finn RD (2014). Pfam: the protein families database. Nucleic acids research.

[58] Gasteiger E (2003). ExPASy: The proteomics server for in-depth protein knowledge and analysis. Nucleic acids research.

[59] Liu RH, Meng JL (2003). [MapDraw: a microsoft excel macro for drawing genetic linkage maps based on given genetic linkage data]. Yi chuan = Hereditas / Zhongguo yi chuan xue hui bian ji.

[60] Krzywinski M (2009). Circos: an information aesthetic for comparative genomics. Genome research.

[61] Zhu H (2012). Unique expression, processing regulation, and regulatory network of peach (Prunus persica) miRNAs. BMC plant biology.

