# Supplementary material for: Global Identification, Classification, and Expression Analysis of MAPKKK genes: Functional Characterization of MdRaf5 Reveals Evolution and Drought-Responsive Profile in Apple
Source: Sci Rep. 2017 Oct 18;7:13511. doi: 10.1038/s41598-017-13627-2 (PMC5647345; doi:10.1038/s41598-017-13627-2)
Supplement: Supplementary file 1 — Supplementary information [file 41598_2017_13627_MOESM1_ESM.pdf]

Title: Global Identification, Classification, and Expression Analysis of MAPKKK genes: Functional Characterization of *MdRaf5* Reveals Evolution and Drought-Responsive Profile in Apple

Authors: Meihong Sun*[1], Yang Xu*[1], Jinguang Huang[1], Zesheng Jiang[1], Huairui Shu[1], Huasen Wang[#2] and Shizhong Zhang[#1]

Affiliation and address:
[1] State Key Laboratory of Crop Biology, Shandong Agricultural University, Tai′an, Shandong 271018, P.R. China
[2] Zhejiang Agriculture & Forestry University, Linan, Zhejiang, 311300, P.R. China.
* Co-first Author: Meihong Sun, Yang Xu.
* The authors contributed equally to this work.

[#] Corresponding author:
Shizhong Zhang
State Key Laboratory of Crop Biology, Shandong Agricultural University, Tai′an, Shandong 271018, P.R. China.
Tel: +86-538-8242364
E-mail: shizhong@sdau.edu.cn

Huasen Wang
Zhejiang Agriculture & Forestry University, Linan, Zhejiang, 311300, P.R. China.
E-mail: whsych66@163.com

Fig.S1

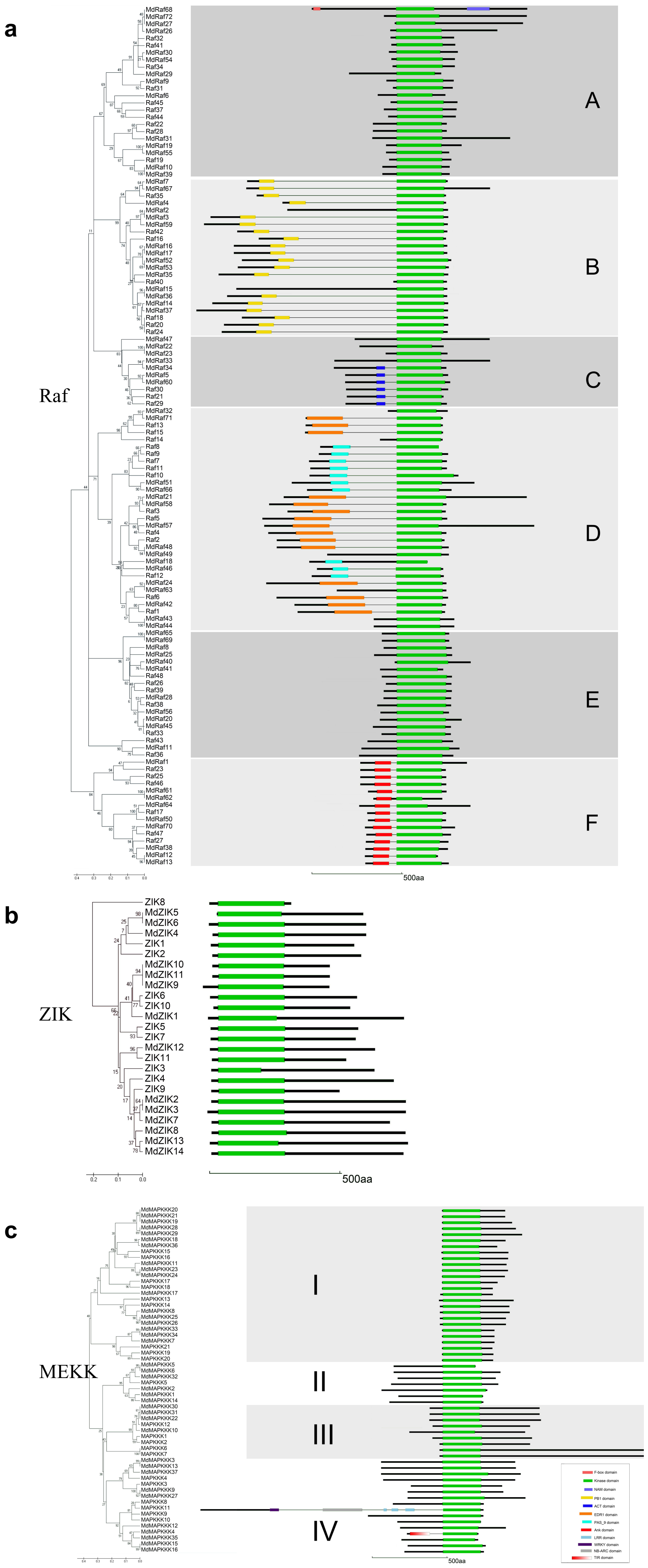

Figure S1. Phylogenetic relationship and protein structure of MAPKKKs in the Arabidopsis and apple. The following parts are shown from left to right. The Phylogenetic tree was constructed according to a complete MAPKKKs protein sequence alignment in the Arabidopsis and apple by the NJ method with bootstrapping analysis (1,000 replicates). (a) The phylogenetic relationship and protein structure of Raf in the Arabidopsis and apple. (b) The phylogenetic relationship and protein structure of ZIK in the Arabidopsis and apple. (c) The phylogenetic relationship and protein structure of MEKK in the Arabidopsis and apple. The scale bar represents 0.05 amino acid substitution in each site. Protein structure: The protein structures were examined by PFAM. The colorful boxes represent the protein domains. The domain abbreviations are: Kinase domain (PF00069); F-box domain (PF00646); NAM domain (PF02365); PB1 domain (PF00564); ACT domain (PF01842); EDR1 domain (PF14381); PAS_9 domain (PF13426); Ank domain (PF00023); LRR domain (PF00560); WRKY domain (PF03106); NB-ARC domain (PF00931); TIR domain (PF01582). The proteins sizes are drawn on bottom.

Fig.S2

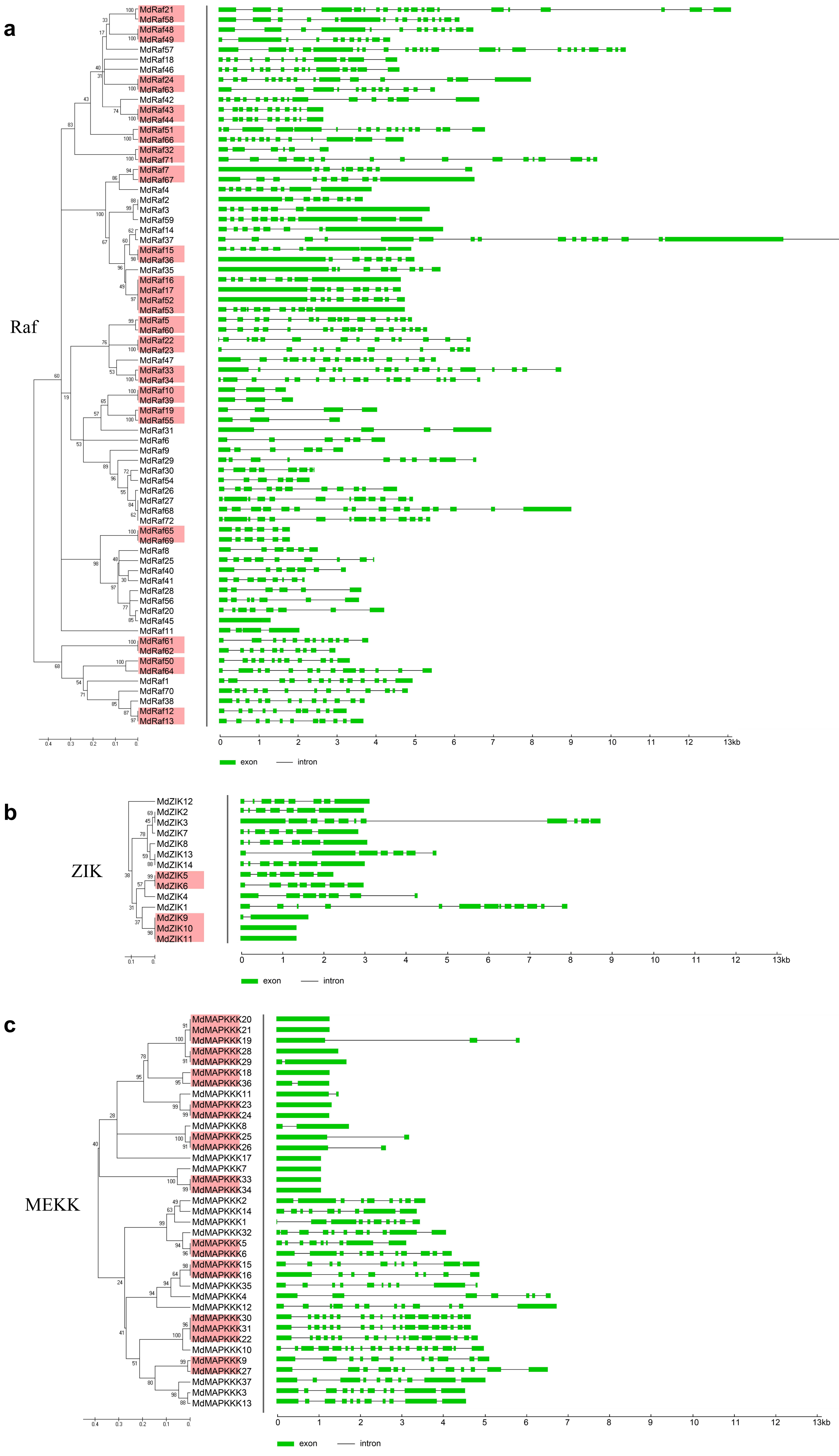

Figure S2. The phylogenetic relationship and the exon/intron structure of apple MAPKKK genes. (a)The phylogenetic relationship and the exon/intron structure of apple Raf genes. (b)The phylogenetic relationship and the exon/intron structure of apple ZIK genes. (c)The phylogenetic relationship and the exon/intron structure of apple MEKK genes. Introns and exons are represented by the black lines and the green boxes. The sizes of exons, the introns and the untranslated regions are drawn on bottom.

Fig.S3

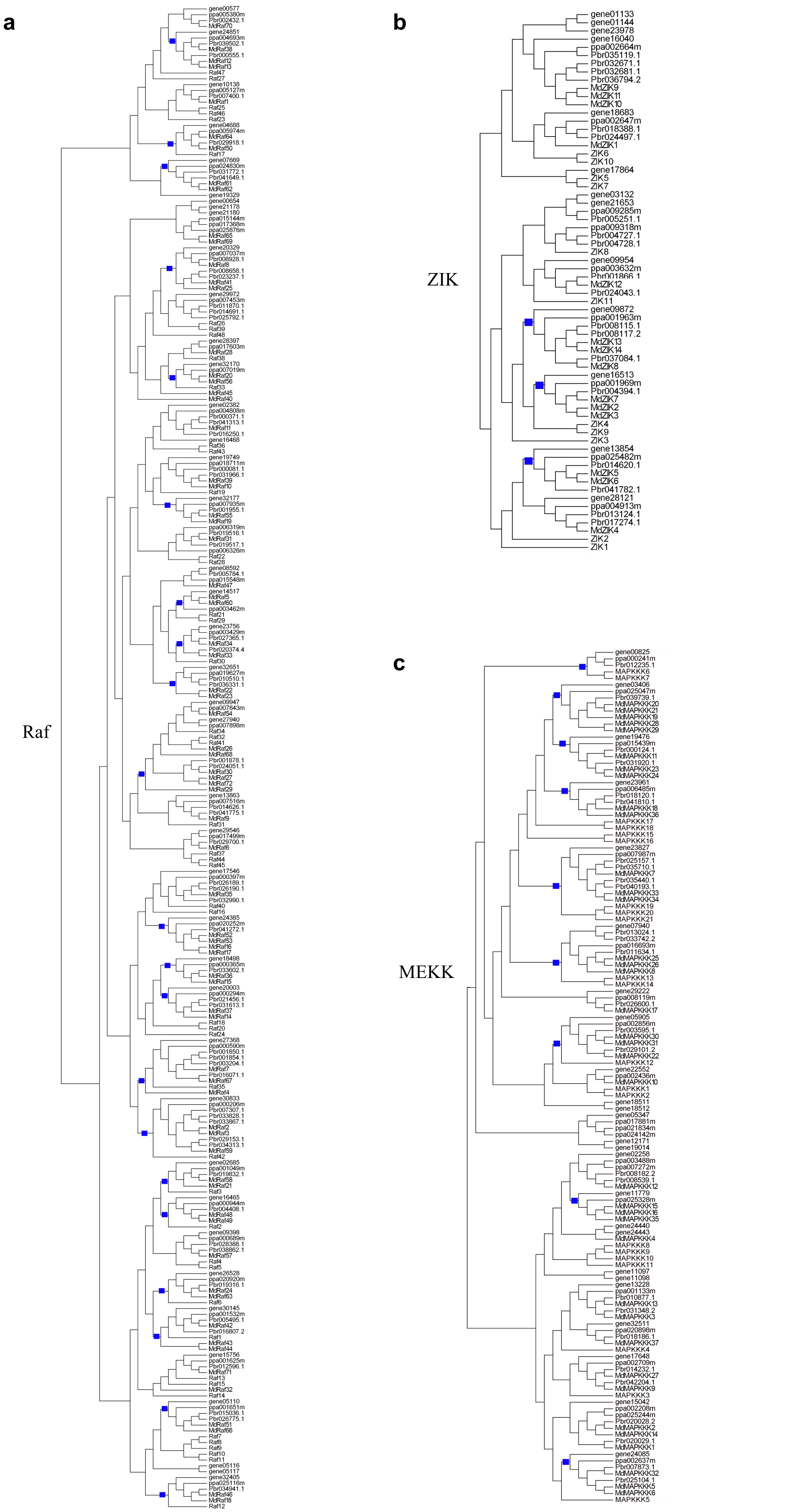

Figure S3. Phylogenetic relationship of MAPKKK genes in apple, peach and pear. (a) Phylogenetic relationship of Raf genes. (b) Phylogenetic relationship of ZIK genes. (c) Phylogenetic relationship of MEKK genes. According to a MdMAPKKK complete protein sequence alignment, the phylogenetic tree was constructed through the neighbor-joining method and with bootstrapping analysis (1,000 replicates). Scale bar represents 0.05 amino acid substitution in each site.

Fig.S4

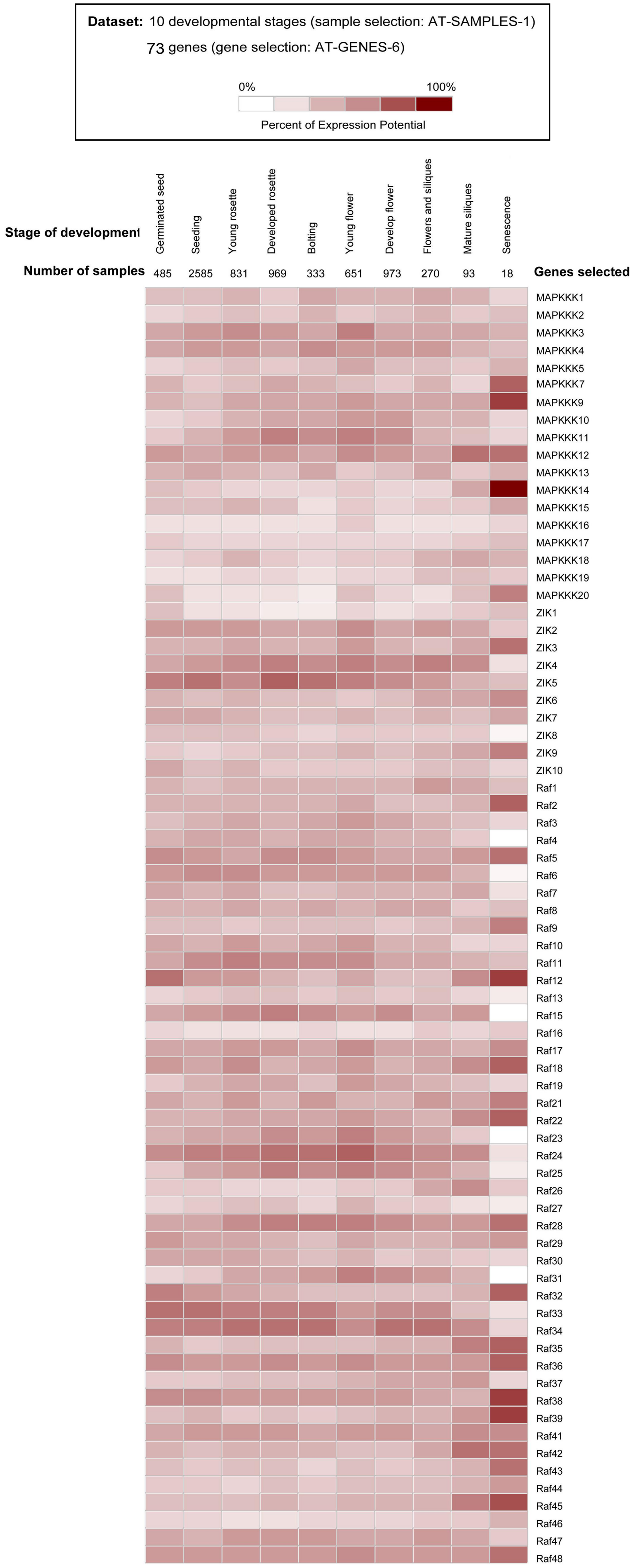

Figure S4. The expression profile of MAPKKK genes in Arabidopsis. The treatment of the deep and light shading represents the relative high or low expressing levels, of the MAPKKK genes development stage in *Arabidopsis*.

Fig.S5

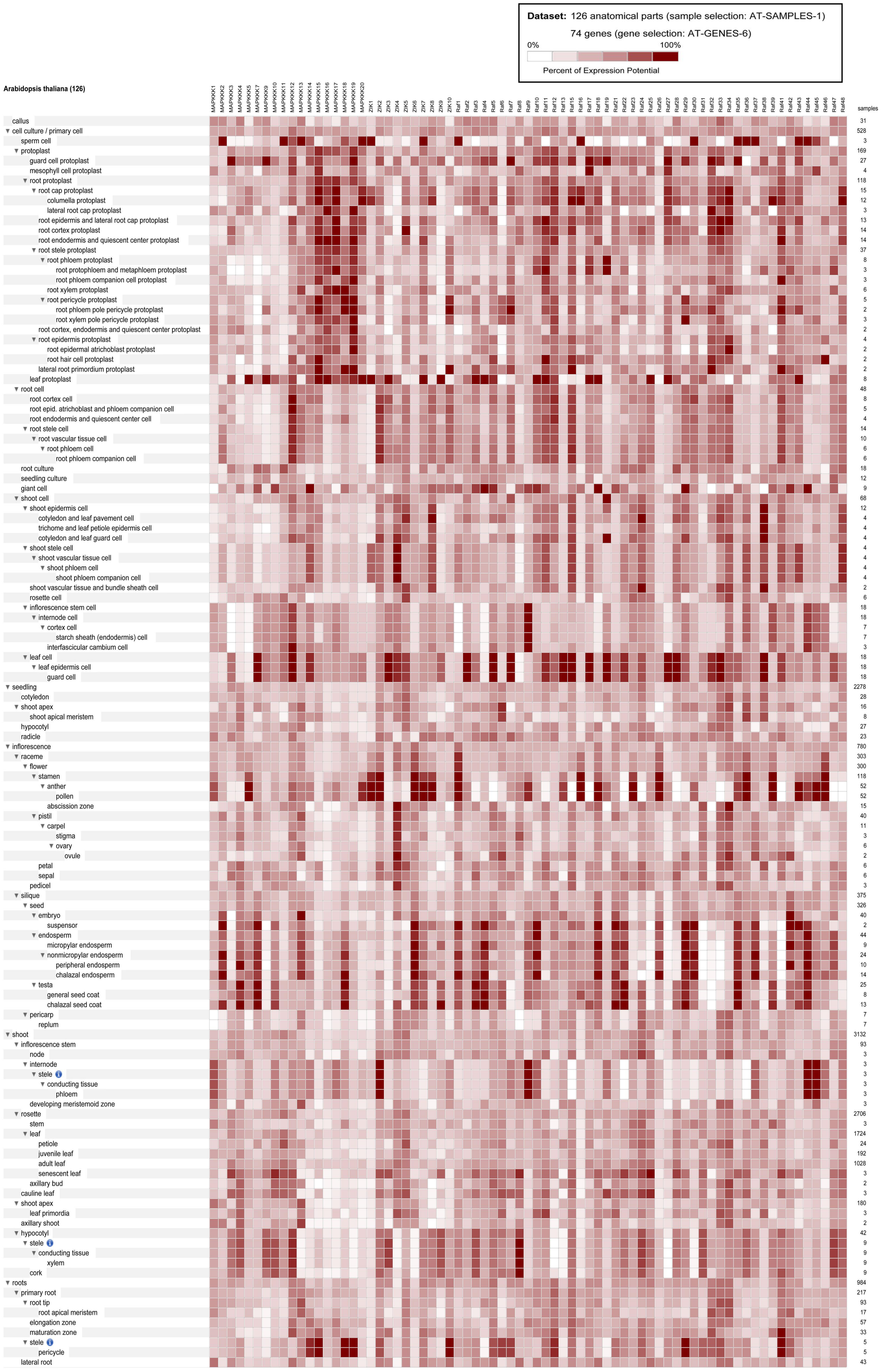

Figure S5. The expression profile of MAPKKK genes in Arabidopsis. The deep and light shading represents the relative high or low expression levels, respectively, of the MAPKKK genes in Arabidopsis tissue.

Fig.S6

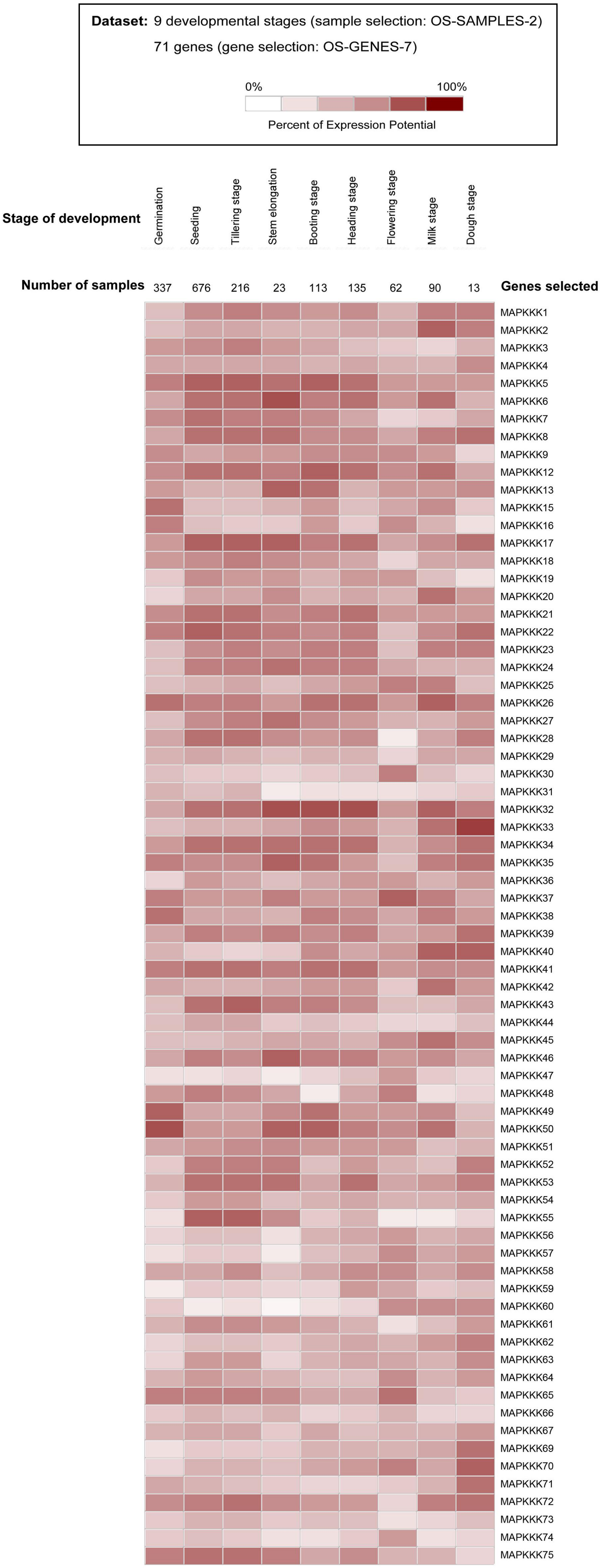

Figure S6. The MAPKKK genes expression profile in rice. The treatment of the deep and light shading represents the relative high or low expressing levels, of the MAPKKK genes development stage in rice.

Fig.S7

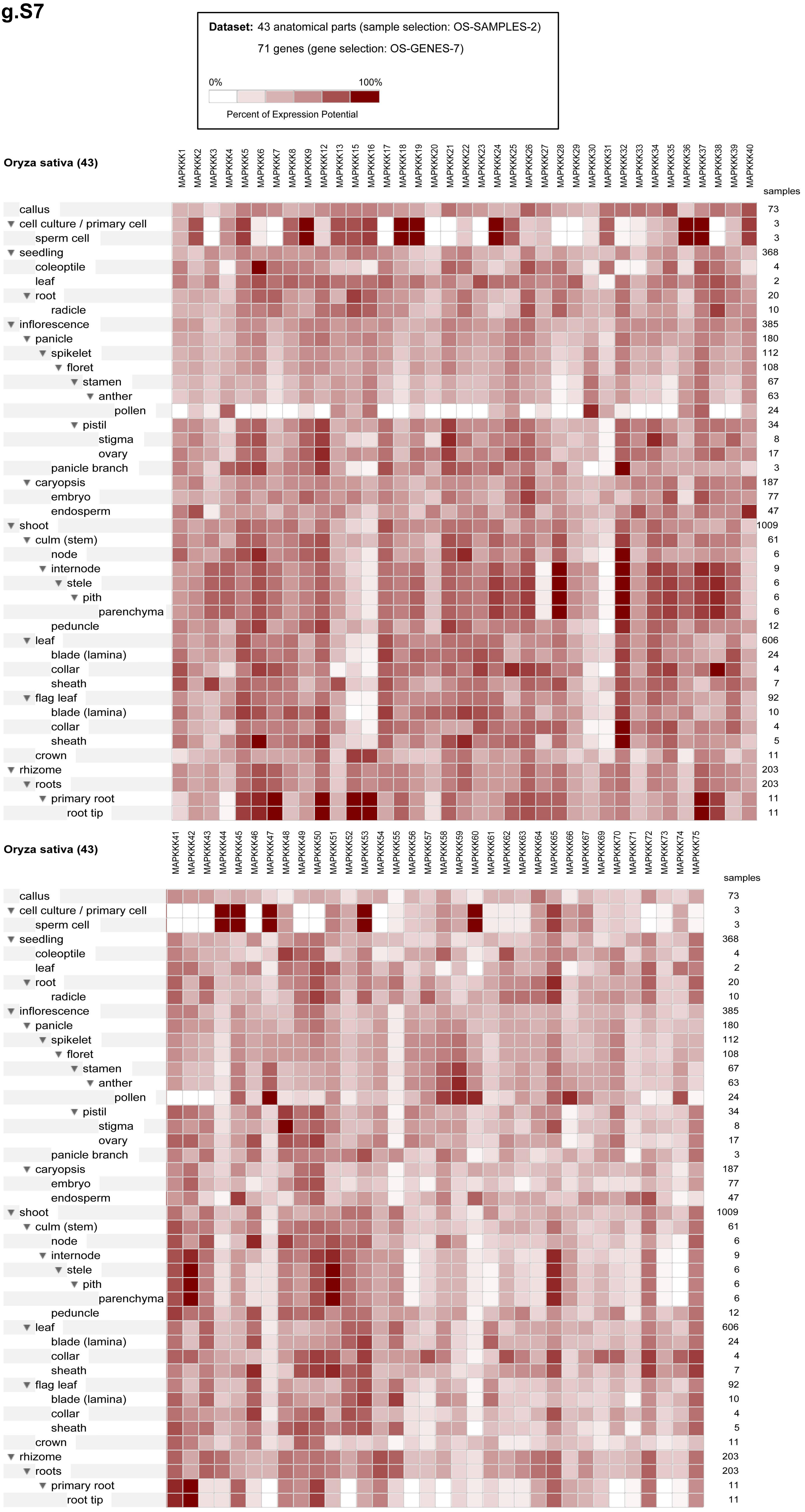

Figure S7. The expression profile of MAPKKK genes in rice. The treatment of deep and light shading represents the relative high or low expressing levels, of the MAPKKK genes in the rice tissue.

Table S1. Genome-wide analysis of MAPKKK genes in apple.

Gene name	Gene model	Genomic position	ORF (bp)	Exon no.	Size(aa)	MW(Da)	pI
MdMAPKKK1	MDP0000220177	chr1:26285627..26289024	1701	10	566	61785.22	8.1
MdMAPKKK2	MDP0000220179	chr1:26292674..26296202	2124	9	707	76175.82	9.31
MdMAPKKK3	MDP0000181878	chr1:27034233..27038699	2697	11	898	96806.59	9.28
MdMAPKKK4	MDP0000295586	chr2:7377206..7383704	1434	7	477	53837.18	5.79
MdMAPKKK5	MDP0000204143	chr2:15458742..15461815	1647	9	548	59939.81	9.15
MdMAPKKK6	MDP0000223670	chr2:15462123..15466270	2148	11	715	78201.34	9.1
MdMAPKKK7	MDP0000187103	chr2:16432317..16433369	1053	1	350	38766.99	5.23
MdMAPKKK8	MDP0000217310	chr3:5805656..5807395	1401	2	466	50733.29	5.32
MdMAPKKK9	MDP0000132521	chr3:6250747..6255784	1923	11	640	69854.51	9.1
MdMAPKKK10	MDP0000295783	chr4:20016291..20021206	2325	19	774	86166.65	6.11
MdMAPKKK11	MDP0000457862	chr5:1145694..1147185	1296	2	431	46789.29	5.5
MdMAPKKK12	MDP0000212585	chr7:18425987..18432622	2127	12	708	78277.37	5.91
MdMAPKKK13	MDP0000314843	chr7:24187234..24191723	2706	11	901	97047.78	9.32
MdMAPKKK14	MDP0000833077	chr8:7957775..7961092	1887	9	628	68347.67	9.35
MdMAPKKK15	MDP0000226096	chr8:28072863..28077668	1569	10	522	58127.8	6.68
MdMAPKKK16	MDP0000167175	chr8:28078248..28083052	1596	8	531	58441.89	6.48
MdMAPKKK17	MDP0000775472	chr8:29185868..29186920	1053	1	350	38276.04	5.47
MdMAPKKK18	MDP0000917158	chr9:7415170..7416444	1275	1	424	46859.98	4.67
MdMAPKKK19	MDP0000313042	chr9:12758444..12764303	1410	3	469	51753.34	5.17
MdMAPKKK20	MDP0000125731	chr9:12771531..12772802	1272	1	423	46821.96	4.64
MdMAPKKK21	MDP0000120204	chr9:12773940..12775211	1272	1	423	46821.96	4.64
MdMAPKKK22	MDP0000225672	chr10:22700727..22705496	2241	17	746	82330.35	7.26
MdMAPKKK23	MDP0000642234	chr10:31955381..31956706	1326	1	441	48883.65	6.23
MdMAPKKK24	MDP0000389544	chr10:31955745..31957007	1263	1	420	46511.59	7.53
MdMAPKKK25	MDP0000431417	chr11:6191070..6194259	1320	2	439	47473.84	4.94
MdMAPKKK26	MDP0000512062	chr11:6200132..6202720	1320	2	439	47473.84	4.94
MdMAPKKK27	MDP0000311835	chr11:6436422..6442849	2370	12	789	86768.53	9.32
MdMAPKKK28	MDP0000267621	chr11:23002516..23004000	1485	1	494	54557.63	4.68
MdMAPKKK29	MDP0000193927	chr11:23007948..23009630	1611	2	536	59110.03	4.67
MdMAPKKK30	MDP0000127586	chr13:18090885..18095490	2211	18	736	81197.96	7.55
MdMAPKKK31	MDP0000197511	chr13:18091046..18095651	2211	18	736	81157.47	7.26
MdMAPKKK32	MDP0000216426	chr15:22955872..22959888	1974	11	657	71633.22	7.55
MdMAPKKK33	MDP0000835932	chr15:23910626..23911678	1053	1	350	38957.99	4.91
MdMAPKKK34	MDP0000784168	chr15:23914447..23915499	1053	1	350	38957.99	4.91
MdMAPKKK35	MDP0000174526	chr15:43889509..43894269	1542	10	513	56565.39	6.81
MdMAPKKK36	MDP0000237405	chr17:8046595..8047861	1113	2	370	40567.63	4.93
MdMAPKKK37	MDP0000168962	chr0:14381113..14386059	2805	10	934	101991.8	9.03
MdRaf1	MDP0000202256	chr2:1201459..1206386	1782	13	593	66877.74	8.62
MdRaf2	MDP0000423001	chr2:1879394..1883074	2682	8	893	99109.39	5.53
MdRaf3	MDP0000516949	chr2:1889287..1894677	4203	8	1400	152235.3	5.22
MdRaf4	MDP0000128251	chr2:3397320..3401221	2733	9	910	100388.8	8.68
MdRaf5	MDP0000223397	chr2:10573575..10578530	1743	16	580	65355.58	5.71
MdRaf6	MDP0000258566	chr2:13315546..13319825	1131	6	376	41874.5	6.24

MdRaf6	MDP0000258566	chr2:13315546..13319825	1131	6	376	41874.5	6.24
MdRaf7	MDP0000160327	chr3:3297289..3303766	3357	9	1118	124210.1	5.47
MdRaf8	MDP0000270013	chr3:30943724..30946234	1137	6	378	42105.98	7.08
MdRaf9	MDP0000627768	chr4:17386638..17389828	1119	6	372	41828.28	8.75
MdRaf10	MDP0000306767	chr5:636894..638610	1125	3	374	42574.15	9.11
MdRaf11	MDP0000313956	chr5:4421693..4423735	1635	4	544	61094.36	9.18
MdRaf12	MDP0000245873	chr5:5896343..5899588	1224	10	407	46208.14	5.47
MdRaf13	MDP0000250081	chr5:5922533..5926204	1398	11	465	53132.72	6.08
MdRaf14	MDP0000778486	chr5:9048140..9053872	3945	8	1314	144793.6	5.43
MdRaf15	MDP0000242830	chr5:10560203..10565155	3528	10	1175	129350	5.24
MdRaf16	MDP0000215190	chr6:3917622..3922308	3567	9	1188	132126.3	6.03
MdRaf17	MDP0000284492	chr6:3924934..3929620	3567	9	1188	132126.3	6.03
MdRaf18	MDP0000136500	chr6:9843471..9848033	1977	12	658	74659.01	8.61
MdRaf19	MDP0000193535	chr6:19752607..19756667	1266	4	421	47744.04	9.38
MdRaf20	MDP0000513685	chr6:19940390..19944590	1365	8	454	50895.12	8.64
MdRaf21	MDP0000311788	chr8:7976741..7989832	4062	19	1353	149584.4	6.33
MdRaf22	MDP0000139057	chr8:21825494..21831962	1407	14	468	52827.03	5.81
MdRaf23	MDP0000483321	chr8:21828009..21834456	1029	10	342	38305.75	5.46
MdRaf24	MDP0000188604	chr8:24296630..24304614	3015	15	1004	111845.9	6.4
MdRaf25	MDP0000164923	chr8:29271674..29275622	1302	9	433	48593.76	8.18
MdRaf26	MDP0000225620	chr9:1446688..1451219	1788	11	595	65839.56	7.32
MdRaf27	MDP0000182722	chr9:1472825..1477764	2142	12	713	79444.05	8.72
MdRaf28	MDP0000231545	chr9:1991422..1995046	1128	6	375	41547.54	6.76
MdRaf29	MDP0000285212	chr9:2688233..2694845	1542	10	513	58399.34	8.67
MdRaf30	MDP0000226571	chr9:15232060..15234512	1155	8	384	43939.18	9.07
MdRaf31	MDP0000236911	chr9:16267743..16274744	2307	4	768	86478.24	7.12
MdRaf32	MDP0000130511	chr9:27051472..27054278	1035	6	344	39511.64	7.04
MdRaf33	MDP0000290680	chr10:7019712..7028502	2604	17	867	97183.15	5.84
MdRaf34	MDP0000223570	chr10:12881981..12888701	1905	17	634	71129.35	6.35
MdRaf35	MDP0000163441	chr10:16782239..16787935	3840	9	1279	140374.1	5.83
MdRaf36	MDP0000288970	chr10:22103769..22108789	3681	8	1226	135582.4	5.48
MdRaf37	MDP0000271642	chr10:24101055..24119000	6114	18	2037	225516.9	6.15
MdRaf38	MDP0000241912	chr10:26522671..26526368	1380	12	459	52482.2	6.55
MdRaf39	MDP0000294384	chr10:32255330..32257228	1125	3	374	42652.55	9.1
MdRaf40	MDP0000169287	chr11:3219225..3222449	1269	7	422	47327.73	8.75
MdRaf41	MDP0000200709	chr11:3406285..3408458	1053	8	350	39521.95	7.08
MdRaf42	MDP0000230308	chr12:1591760..1598416	2532	15	843	92844.39	5.8
MdRaf43	MDP0000126707	chr12:1685229..1687902	1350	11	449	49959.58	7.72
MdRaf44	MDP0000151497	chr12:1687561..1690234	1350	11	449	49954.98	7.72
MdRaf45	MDP0000615514	chr12:10329691..10331003	1302	1	433	47563.29	7.52
MdRaf46	MDP0000173360	chr12:20192434..20197056	2109	15	702	79313.97	7.05
MdRaf47	MDP0000253129	chr12:20858085..20863668	2256	16	751	83468.8	5.96
MdRaf48	MDP0000176409	chr12:29794837..29801346	2877	13	958	105223.3	6.17
MdRaf49	MDP0000785379	chr12:29829897..29834270	2028	11	675	75225.63	7

MdRaf50	MDP0000934762	chr13:4231756..4235078	1302	11	433	48762.35	6.94
MdRaf51	MDP0000165867	chr13:13708625..13715423	3081	17	1026	114425.6	9
MdRaf52	MDP0000196280	chr13:31459388..31464167	3501	10	1166	130721.1	5.83
MdRaf53	MDP0000147195	chr13:31468138..31472917	3528	11	1175	131418.6	5.85
MdRaf54	MDP0000329851	chr14:23296610..23298954	1062	7	353	40013.51	7.64
MdRaf55	MDP0000280463	chr14:24610560..24613659	1056	3	351	39812.1	8.32
MdRaf56	MDP0000165481	chr14:24714078..24717633	1143	7	380	42506.47	6.33
MdRaf57	MDP0000296958	chr14:28292656..28303065	4515	24	1504	166228.1	5.97
MdRaf58	MDP0000225231	chr15:859873..866023	2964	15	987	108668.1	5.45
MdRaf59	MDP0000222723	chr15:8653511..8658705	4077	11	1358	147948.5	5.3
MdRaf60	MDP0000170853	chr15:17489514..17494859	1779	16	592	67085.82	5.83
MdRaf61	MDP0000270104	chr15:37272478..37276269	1314	12	437	49556.99	7.15
MdRaf62	MDP0000163771	chr15:37273277..37276235	1152	10	383	43259.7	6.73
MdRaf63	MDP0000682893	chr15:38514953..38520485	1833	12	610	67458.97	6.01
MdRaf64	MDP0000320298	chr16:2794575..2799983	1863	13	620	70950.74	9.46
MdRaf65	MDP0000191774	chr16:4289219..4291018	1125	6	374	42104.66	8.88
MdRaf66	MDP0000865313	chr16:8616834..8621556	2415	13	804	89184.62	6.59
MdRaf67	MDP0000240766	chr16:20051044..20057578	4071	10	1356	151298.8	5.62
MdRaf68	MDP0000283975	chr17:1417327..1426290	3606	16	1201	135662.3	7.68
MdRaf69	MDP0000168375	chr17:7886126..7887925	1125	6	374	42121.5	8.95
MdRaf70	MDP0000142826	chr17:12220260..12225062	1509	13	502	57382.24	5.83
MdRaf71	MDP0000051653	chr17:23244891..23254563	2442	16	813	91859.98	6.1
MdRaf72	MDP0000187308	chr0:68327503..68332871	2388	14	795	88548.8	9.04
MdZIK1	MDP0000245016	chr1:11484792..11492726	2259	13	752	83882.06	5.84
MdZIK2	MDP0000899699	chr4:21090387..21093384	2238	7	745	84964.05	5.03
MdZIK3	MDP0000311885	chr4:21100781..21109524	3246	11	1081	121869.4	5.76
MdZIK4	MDP0000457389	chr11:4762008..4766306	1767	7	588	66535.79	6.14
MdZIK5	MDP0000574890	chr12:25854565..25856812	1686	6	561	64168.92	5.22
MdZIK6	MDP0000274290	chr12:25859782..25862768	1815	7	604	68832.3	5.45
MdZIK7	MDP0000160172	chr12:29916969..29919823	2055	7	684	77436.66	5.2
MdZIK8	MDP0000152621	chr13:18719788..18722861	2235	7	744	84807.97	5.35
MdZIK9	MDP0000147900	chr13:20585304..20586949	1458	2	485	55014.4	5.75
MdZIK10	MDP0000195409	chr13:20587688..20589043	1356	1	451	51295.42	5.21
MdZIK11	MDP0000281707	chr13:20588855..20590210	1356	1	451	51258.96	5.16
MdZIK12	MDP0000184651	chr14:23252025..23255140	1899	8	632	70480.72	4.87
MdZIK13	MDP0000589319	chr16:14889804..14894557	2283	7	760	86802.37	5.21
MdZIK14	MDP0000197707	chr16:14901991..14905007	2223	7	740	84319.38	5.21

Table S1. Genome-wide analysis of MAPKKK genes in apple. Gene name and position of 123 MAPKKKs. ORF length, numbers of amino acids are analysed in GDR database (Genome Database for Rosaceae: http://www.rosaceae.org/). molecular weight, and isoelectric points (pIs) are analysed on ExPASy Proteomics Server (http://expasy.org/).

Table S2. The number of MAPKKK genes in different plants.

Species	Raf	ZIK	MEKK	Total
Arabidopsis	48	11	21	80
rice	43	10	22	75
maize	46	6	22	74
apple	72	14	37	123
peach	39	9	21	69
strawberry	41	13	25	79
pear	58	19	27	104

Table S2: The number of MAPKKK genes in Arabidopsis, rice, maize, apple, peach, strawberry and pear.

Table S3. Ka/Ks analysis and synteny blocks for the duplicated gene pairs in MAPKKK gene family between Arabidopsis and apple.

BLOCK_SCORE	E_VALUE	LOCUS _At	Gene_At	LOCUS_Md	Gene_Md	Ka	Ks	Ka/Ks
401	0	AT3G59830	*Raf46*	MDP0000202256	*MdRaf1*	0.1615	2.1123	0.08
203	4.00E-124	AT5G66850	*MAPKKK5*	MDP0000204143	*MdMAPKKK5*	0.3508	1.6367	0.21
236	3.00E-141	AT5G01850	*Raf31*	MDP0000627768	*MdRaf9*	0.1617	-1	-0.16
462	8.00E-156	AT1G54960	*MAPKKK2*	MDP0000295783	*MdMAPKKK10*	0.3092	1.6631	0.19
264	0	AT2G35050	*Raf24*	MDP0000778486	*MdRaf14*	0.453	1.6169	0.28
634	0	AT1G79570	*Raf20*	MDP0000242830	*MdRaf15*	0.4731	1.5105	0.31
347	0	AT1G16270	*Raf18*	MDP0000242830	*MdRaf15*	0.4451	1.5542	0.29
529	0	AT4G24480	*Raf6*	MDP0000188604	*MdRaf24*	0.3015	1.6524	0.18
336	2.00E-83	AT1G05100	*MAPKKK18*	MDP0000917158	*MdMAPKKK18*	0.3973	1.8411	0.22
521	2.00E-83	AT2G32510	*MAPKKK17*	MDP0000917158	*MdMAPKKK18*	0.4985	1.8463	0.27
292	0	AT4G38470	*Raf30*	MDP0000223570	*MdRaf34*	0.191	1.3413	0.14
248	0	AT1G79570	*Raf20*	MDP0000288970	*MdRaf36*	0.4947	1.9351	0.26
326	0	AT2G35050	*Raf24*	MDP0000271642	*MdRaf37*	0.4331	1.8081	0.24
315	9.00E-178	AT1G62400	*Raf19*	MDP0000294384	*MdRaf39*	0.0883	-1	-0.09
274	0	AT5G58350	*ZIK2*	MDP0000457389	*MdZIK4*	0.2794	2.5291	0.11
246	5.00E-103	AT1G07150	*MAPKKK13*	MDP0000431417	*MdMAPKKK25*	0.429	1.5051	0.29
240	2.00E-102	AT2G30040	*MAPKKK14*	MDP0000431417	*MdMAPKKK25*	0.4776	1.9459	0.25
302	2.00E-84	AT4G26890	*MAPKKK16*	MDP0000267621	*MdMAPKKK28*	0.4701	2.8285	0.17
1003	1.00E-99	AT5G55090	*MAPKKK15*	MDP0000267621	*MdMAPKKK28*	0.4376	2.4987	0.18
380	3.00E-149	AT4G35780	*Raf29*	MDP0000253129	*MdRaf47*	0.437	3.7756	0.12
203	2.00E-157	AT3G51630	*ZIK1*	MDP0000574890	*MdZIK5*	0.2893	-1	-0.29
1001	0	AT3G04910	*ZIK4*	MDP0000160172	*MdZIK7*	0.278	-1	-0.28
272	0	AT3G06620	*Raf7*	MDP0000165867	*MdRaf51*	0.2369	1.3607	0.17
272	0	AT5G49470	*Raf10*	MDP0000165867	*MdRaf51*	0.1744	1.3114	0.13
927	4.00E-180	AT3G06030	*MAPKKK12*	MDP0000127586	*MdMAPKKK30*	0.3227	1.7877	0.18
761	1.00E-175	AT3G22420	*ZIK3*	MDP0000152621	*MdZIK8*	0.2628	1.8682	0.14
416	0	AT2G17700	*Raf21*	MDP0000170853	*MdRaf60*	0.1958	1.4503	0.14
1535	0	AT4G35780	*Raf29*	MDP0000170853	*MdRaf60*	0.1698	1.8094	0.09
330	1.00E-94	AT3G50310	*MAPKKK20*	MDP0000835932	*MdMAPKKK33*	0.3688	2.7507	0.13
500	7.00E-95	AT4G36950	*MAPKKK21*	MDP0000835932	*MdMAPKKK33*	0.3784	-1	-0.38
630	1.00E-98	AT5G67080	*MAPKKK19*	MDP0000835932	*MdMAPKKK33*	0.3364	2.5023	0.13
1487	0	AT1G14000	*Raf17*	MDP0000320298	*MdRaf64*	0.1344	2.679	0.05
353	0	AT3G06620	*Raf7*	MDP0000865313	*MdRaf66*	0.2497	1.3869	0.18
235	1.00E-172	AT5G49470	*Raf10*	MDP0000865313	*MdRaf66*	0.1886	1.335	0.14
845	3.00E-168	AT3G22420	*ZIK3*	MDP0000589319	*MdZIK13*	0.27	2.1039	0.13
249	0	AT5G57610	*Raf35*	MDP0000240766	*MdRaf67*	0.3074	1.5669	0.2
463	2.00E-75	AT2G32510	*MAPKKK17*	MDP0000237405	*MdMAPKKK36*	0.4914	3.2311	0.15
223	1.00E-160	AT3G58760	*Raf47*	MDP0000142826	*MdRaf70*	0.2596	1.4596	0.18

Table S3: The Ks and Ka were calculated by the DnaSP v5.0 software (DNA polymorphism analysis).

Table S4. The partial RNA-sequence of differentially expressed genes between CK and D.

gene name	geneID	CK-Expression	D-Expression	CK-FPKM	D-FPKM	log2 Ratio (D/CK)	Up-Down-Regulation(D/CK)
Raf5	MDP0000223397	0.5	7	0.22	3.31	3.911255788	Up
MAPKKK4	MDP0000295586	1	8	0.73	6.31	3.111671636	Up
Raf57	MDP0000296958	1.45	10.18	0.53	4.07	2.94096453	Up
Raf31	MDP0000236911	7.02	41.44	2.97	18.95	2.673663012	Up
Raf51	MDP0000165867	2	11.19	0.19	1.17	2.622437206	Up
Raf7	MDP0000160327	2	11	0.54	3.19	2.562525112	Up
Raf9	MDP0000627768	1.14	5.76	1.04	5.68	2.449307401	Up
ZIK1	MDP0000245016	1	4.98	0.35	1.89	2.432959407	Up
Raf6	MDP0000258566	1.94	5.37	1.57	4.71	1.584962501	Up
Raf41	MDP0000200709	4.76	13.22	1.55	4.65	1.584962501	Up
Raf59	MDP0000222723	2	4	4.45	9.62	1.112231558	Up
Raf19	MDP0000193535	5	10	1.67	3.61	1.112150734	Up
ZIK8	MDP0000152621	6	10	6.88	12.4	0.849859651	Up
MAPKKK20	MDP0000125731	4	6.63	2.16	3.88	0.84502534	Up
Raf1	MDP0000202256	2.07	3.06	0.37	0.6	0.69743723	Up
MAPKKK35	MDP0000174526	2	3	1.48	2.4	0.69743723	Up
Raf13	MDP0000250081	13	18	4.78	7.16	0.582948969	Up
Raf20	MDP0000513685	17	23	4.69	6.87	0.550722176	Up
Raf12	MDP0000245873	3	4	0.46	0.66	0.520832163	Up
ZIK11	MDP0000281707	1.69	2	0.15	0.19	0.341036918	Up
ZIK9	MDP0000147900	11.99	14.04	15.2	19.25	0.340787122	Up
Raf68	MDP0000283975	7	8	5.83	7.21	0.306503376	Up
Raf50	MDP0000934762	10.44	11.95	3	3.71	0.306456686	Up
Raf58	MDP0000225231	15	17	2.36	2.9	0.297266041	Up
ZIK14	MDP0000197707	89.52	101.63	12.16	14.94	0.297036919	Up
ZIK5	MDP0000574890	99	112	14.22	17.4	0.291165841	Up
Raf10	MDP0000306767	7.36	8.31	2.06	2.52	0.290779396	Up
MAPKKK13	MDP0000314843	8	9	2.3	2.8	0.283792966	Up
MAPKKK34	MDP0000784168	32	36	7.73	9.41	0.283726309	Up
MAPKKK21	MDP0000120204	1	1	0.51	0.55	0.108934372	Up
Raf14	MDP0000778486	1	1	1.02	1.1	0.108934372	Up
Raf21	MDP0000311788	1	1.01	0.51	0.55	0.108934372	Up
Raf30	MDP0000226571	6	6.01	1.02	1.1	0.108934372	Up
Raf24	MDP0000188604	29.79	27.83	49.16	49.69	0.015470626	Up
ZIK7	MDP0000160172	15	14	15.44	15.59	0.013948175	Up
ZIK10	MDP0000195409	29	27	27.77	27.98	0.010868785	Up
Raf64	MDP0000320298	9	6	6.01	4.33	-0.472997966	Down
MAPKKK7	MDP0000187103	6	4	3.22	2.32	-0.472935883	Down
Raf28	MDP0000231545	25	19	6.04	4.97	-0.281302698	Down
Raf66	MDP0000865313	55.17	41.99	5.05	4.16	-0.279699859	Down
Raf72	MDP0000187308	67	51	18.9	15.57	-0.27961729	Down
Raf25	MDP0000164923	11	8.39	24.05	19.85	-0.276897887	Down
ZIK3	MDP0000311885	38.01	29	38.66	31.91	-0.276833029	Down
Raf54	MDP0000329851	4	3	0.23	0.19	-0.275634443	Down
Raf46	MDP0000173360	2.79	2.09	0.46	0.38	-0.275634443	Down
MAPKKK36	MDP0000237405	26.4	20.2	10.36	8.57	-0.273656894	Down

Table S4. The partial RNA-sequence of differentially expressed genes between CK and D. CK: RNA of *Malus hupehensis* (*Malus hupehesis* (Pamp.) Rehd. *var. pinyiensis* under nomal condition. D: RNA of *Malus hupehensis* (*Malus hupehesis* (Pamp.) Rehd. *var. pinyiensis* after drought stress treatment.

Table S5. Primers used in this paper.

Gene name	Forward primer (5'-3')	Reverse primer (5'-3')
MdZIK1	CTAACCAGAGTCTGAAAGCGACAA	TACAGAGCAACACCCGCAAA
MdMAPKKK4	TTACAGATCATCTATACCGTGCCT	TACAATGCCAACAGAACCAA
MdMAPKKK7	ACCAGCCCATCTTCTCACCTTCC	TCATCGCCGCCAATCCTCAT
MdRaf5	TGCCTTTGAAGTTCGCTCTG	TCATCCCTTGTTTGATTGTCCT
MdRaf7	GATGCTCTTGTCTCGGTTGTG	TACCCTCGCTGCTTGGATTA
MdRaf9	TCGTCCAAATCCATTAGAACTCC	TGATCTGGCTGAACGTAGGC
MdRaf31	GCAAGGCTTCTACCACAAACTC	TGTTAGGGCGGACACTCTTATT
MdRaf41	TGTTGAGTTGGTGGACGCCTAG	ATGCCGCCTCCCTTGCTATT
MdRaf51	AAGCAGTAGCGCCGGACC	CTCGCCCATTTCCTTTCCAT
MdRaf57	AACCGCCTCATTCTCCGACAC	ACGCCGATCCTCTACCCATT
MdRaf59	ATTTCATCCGCTGCTCCTTA	CATCCTCCCGTGATTTGTCTC
MdRaf64	TCTTTCCGGTGATAGATTGGTG	TTGGCTGCTTCATAAGGCTCGT
18s rRNA	ACACGGGGAGGTAGTGACAA	CCTCCAATGGATCCTCGTTA

Table S5. Primers for making the gene expression analysis by qRT-PCR.
